# Critical summer foraging tradeoffs in a subarctic ungulate

**DOI:** 10.1002/ece3.8349

**Published:** 2021-12-06

**Authors:** Libby Ehlers, Gabrielle Coulombe, Jim Herriges, Torsten Bentzen, Michael Suitor, Kyle Joly, Mark Hebblewhite

**Affiliations:** ^1^ Wildlife Biology Program Department of Ecosystem and Conservation Sciences University of Montana Missoula Montana USA; ^2^ Bureau of Land Management Fairbanks Alaska USA; ^3^ Alaska Department of Fish and Game Fairbanks Alaska USA; ^4^ Yukon Government Dawson City Yukon Territory Canada; ^5^ National Park Service Yukon‐Charley Rivers National Preserve Fairbanks Alaska USA

**Keywords:** animal‐borne video cameras, behavior patterns, caribou, citizen‐science, insect harassment, summer diet

## Abstract

Summer diets are crucial for large herbivores in the subarctic and are affected by weather, harassment from insects and a variety of environmental changes linked to climate. Yet, understanding foraging behavior and diet of large herbivores is challenging in the subarctic because of their remote ranges. We used GPS video‐camera collars to observe behaviors and summer diets of the migratory Fortymile Caribou Herd (*Rangifer tarandus granti*) across Alaska, USA and the Yukon, Canada. First, we characterized caribou behavior. Second, we tested if videos could be used to quantify changes in the probability of eating events. Third, we estimated summer diets at the finest taxonomic resolution possible through videos. Finally, we compared summer diet estimates from video collars to microhistological analysis of fecal pellets. We classified 18,134 videos from 30 female caribou over two summers (2018 and 2019). Caribou behaviors included eating (mean = 43.5%), ruminating (25.6%), travelling (14.0%), stationary awake (11.3%) and napping (5.1%). Eating was restricted by insect harassment. We classified forage(s) consumed in 5,549 videos where diet composition (monthly) highlighted a strong tradeoff between lichens and shrubs; shrubs dominated diets in June and July when lichen use declined. We identified 63 species, 70 genus and 33 family groups of summer forages from videos. After adjusting for digestibility, monthly estimates of diet composition were strongly correlated at the scale of the forage functional type (i.e., forage groups composed of forbs, graminoids, mosses, shrubs and lichens; *r *= 0.79, *p* < .01). Using video collars, we identified (1) a pronounced tradeoff in summer foraging between lichens and shrubs and (2) the costs of insect harassment on eating. Understanding caribou foraging ecology is needed to plan for their long‐term conservation across the circumpolar north, and video collars can provide a powerful approach across remote regions.

## Graphical abstract

Summer diets are crucial for subarctic herbivores and are affected by weather, insect harassment and a variety of environmental changes linked to climate. We used GPS video‐camera collars to observe behavioral patterns and summer diets of migratory caribou (*Rangifer tarandus granti*) across Alaska, USA and Yukon, Canada. Using video collars, we identified (1) a pronounced tradeoff in foraging between lichens and shrubs and (2) the costs of insect harassment on eating in summer.

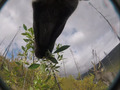

## 1 INTRODUCTION

Climate change in the arctic and subarctic (hereafter, arctic) region is unfolding faster than anywhere else on Earth, resulting in alterations of ecosystem structure and function (Box et al., 2019; Hinzman et al., 2005; IPCC, 2014). Vegetation communities are experiencing abrupt and lasting changes resulting from warming temperatures, increased precipitation and more frequent and severe wildfires (Berner et al., 2020; Loranty et al., 2016; Myers‐Smith et al., 2011; Walker et al., 2006; Wang et al., 2020). Some plant functional types, like shrubs, are expanding their distribution in response to warming temperatures and increased precipitation (i.e., rain) and outcompeting previously dominant functional groups (lichen; Berner et al., 2018; Myers‐Smith et al., 2011).

Changes in vegetation communities are expected to affect ecological carrying capacity through changes to the availability and timing of forage resources (e.g., phenology; Post & Forchhammer, 2008) for herbivores across the circumpolar north (Joly et al., 2012; Post, 2013; Yu et al., 2017). Changing vegetation directly alters the composition, biomass and quality of available forages for large herbivores (Rickbeil et al., 2018; Stark et al., 2021; Zamin et al., 2017). For migratory caribou (e.g., *Rangifer tarandus granti*), the increasing frequency of wildfires is also burning more winter taiga range, removing old‐growth forest bearing lichen, their major forage in winter (Gustine et al., 2014; Joly et al., 2012; Russell, 2018). Warming temperatures also promote insect abundance and activity, forcing caribou to spend less time feeding and more energy on avoidance behaviors (Joly et al., 2020; Weladji et al., 2003; Witter, Johnson, Croft, Gunn, & Gillingham, 2012; Witter, Johnson, Croft, Gunn, & Poirier, 2012).

Previous studies have demonstrated the key role of summer nutrition, especially for arctic ungulates who experience short growing seasons (Barboza et al., 2009; Cook et al., 2004; Shively et al., 2019). Following the forage maturation hypothesis for large herbivores (Fryxell, 1991; Hebblewhite et al., 2008), caribou transition from a diet dominated by low‐quality lichen (winter) to a diet dominated by higher‐quality green vegetation (i.e., graminoids and shrubs) to meet the digestible energy and protein requirements for fetal growth (spring) and lactation (summer; Barboza et al., 2018; Crête & Huot, 1993; Denryter et al., 2020). However, caribou experience nutritional deficiencies due to reproductive costs of lactation and inadequate nutrition for energetic demands in many land cover types in boreal forests (Denryter et al., 2018). Further supporting the nutritional deficiency hypothesis, researchers have shown the highest rates of natural adult mortality for caribou in July and August (Cook et al., 2021; Gurarie et al., 2019; McLoughlin et al., 2003). Thus, identifying tradeoffs between foraging for high‐quality foods and behaviors that inhibit eating, like those resulting from insect harassment and movement, are key to understanding nutritional implications for caribou during summer.

Observational studies of caribou have shown insect harassment reduces the time caribou spent foraging in summer and increases energy expenditures (e.g., movement) that could result in consequences for body weight and thus, reproduction, calf recruitment and survival (Colman et al., 2003; Toupin et al., 1996; Witter, Johnson, Croft, Gunn, & Gillingham, 2012; Witter, Johnson, Croft, Gunn, & Poirier, 2012). Therefore, climate change has the potential to increase both the benefits of foraging, by increasing the availability of high‐quality foods like shrubs, and the costs, through changes to energy budgets from insect harassment. However, measuring foraging ecology of remote caribou in the Arctic remains challenging.

Animal‐borne video cameras provide an exciting opportunity to study large herbivore nutritional ecology especially in remote regions. Animal‐borne video cameras have improved our understanding of foraging ecology for marine, avian and terrestrial species (Kane & Zamani, 2014; Lavelle et al., 2015; Seminoff et al., 2006). Large herbivores are unique in that they spend a great deal of their time foraging, upwards of 14 h every day (e.g., Sukumar, 1989). Animal‐borne cameras have recently been applied to large herbivores across remote regions of Mongolia and Canada (Kaczensky et al., 2019; Vuillaume et al., 2021). Previous studies using video collars have measured foraging and diet, grooming and reproduction across cervids (e.g., Lavelle et al., 2015; Thompson et al., 2012; Viejou et al., 2018). One challenge with any new method, such as animal‐borne video collars, is the calibration with existing methods, for example, to study diet. Previous studies used a variety of diet methods including behavioral observations in the wild (Fortin et al., 2004; Schaller, 1998), captive and/or tame animals (Shipley et al., 1999), harvested animals (Helle & Tarvainen, 1984), stomach diet analyses (Skoog, 1956) and fecal diet analyses (Russell et al., 1993). These diverse methods measure diet at different stages in the foraging process, that is, intake rate (behavioral observations of foraging), in vivo (stomach) or following digestion (fecal samples). They also use different metrics, such as percent composition, frequency, number of bites or intake rate in grams/bite (Robbins et al., 1987; Thompson & Barboza, 2014). Thus, comparing diet estimates from different methods is challenging. Many previous methods, including observations and fecal diet sampling, and newer methods like metagenomics are often limited by sample sizes and are costly to implement in remote arctic regions. Animal‐borne camera collars can, however, provide finer‐scale details of foraging behavior and diet for remote ungulates (e.g., Kaczensky et al., 2019; Thompson et al., 2015; Viejou et al., 2018).

We used animal‐borne GPS video‐camera collars (hereafter, “video collars”) to study behavior and diets of a migratory population of caribou in the subarctic during spring and summer. Caribou are an important cultural, socioeconomic and ecological resource across the circumpolar north (Hummel & Ray, 2008). We focused on adult female caribou during summer because females drive population dynamics (Cook et al., 2021; Roff, 1992). The Fortymile Caribou Herd in central Alaska, USA and Yukon, Canada, is a population that has undergone intensive management for over 50 years (Gronquist et al., 2005; Macdonald et al., 2009). Recent population growth of the Fortymile Caribou Herd (Boertje et al., 2017) has led to questions about deteriorating range conditions and food limitation, for which there is growing evidence for migratory caribou (Bergerud et al., 2008; Crête & Huot, 1993; Schaefer et al., 2016). Due to this, understanding foraging behaviors and summer diets of caribou remains central for managing migratory populations around the globe (Video 1).

**VIDEO 1:** This 2‐min compilation video highlights behaviors and diet items for the migratory Fortymile Caribou Herd in Alaska, USA and Yukon, Canada. From May 10–September 11 (2018 & 2019), GPS video‐camera collars recorded a 9‐s video and GPS location every 20 min during daylight hours. We first used citizen scientists to classify caribou behavior into states of eating, ruminating, travelling, stationary awake, napping and other. For videos classified as ‘eating’, we then used skilled observers to identify forages consumed by caribou during the summer months.

Using videos collected from collars, we first characterized behavioral activities of caribou and quantified insect avoidance behaviors, while considering individual variation among caribou, and tradeoffs between eating and insect avoidance behaviors. To test for individual variation, we also tested for differences in behavioral activities among individual caribou to understand individual‐level variability in behavior. Second, we tested if insect avoidance behaviors reduced the time caribou spent eating (Colman et al., 2003). We predicted the already short summer foraging period would be further restricted by insect harassment. Third, we estimated diet at two levels of taxonomic resolution, the forage functional type (i.e., plants like forbs and shrubs, plus lichen and mushrooms) and the finest taxonomic resolution “species, genera or family” obtained from videos. In the context of the forage maturation hypothesis (Fryxell, 1991), we predicted caribou would switch from a lichen‐based diet in late spring to one of higher protein, green vegetation in summer, ostensibly to replenish protein and fat reserves. We then expected caribou to return to lichen in autumn with the senescence of green vegetation. Finally, we compared diet estimates from video collars to results from fecal pellet microhistology (Dearden et al., 1975) for the Fortymile Caribou Herd, after adjusting for plant digestibility. Addressing our research questions required data classification from video collars, citizen‐science volunteer training, data management and coordination with trained botanists specialized in arctic species to classify plants consumed by caribou. We summarize our protocols and data processing steps (Box 1, Appendix A) because of the growing interest in the application of video collars for arctic wildlife.

BOX 1 Flow chart of our data collection process using caribou video collars. We excluded video recordings that malfunctioned were shorter than 8 s and confirmed videos recorded on schedule for the duration of the study for each caribou. Using R, we created folders of randomly selected videos (with an equal number of videos per study animal). To improve efficiency, we classified videos using two phases. In the first phase (in blue), volunteer observers (citizen scientists) viewed videos to identify caribou behaviors and other supplemental information (see Appendix A). This first phase required approximately 2 min of time per observer to classify a one 9‐s video from caribou. In the second phase (in green), botanists who were specialized in arctic flora viewed videos classified as eating from the first phase to identify forage items consumed by caribou. Botanists identified forages to the most refined taxonomic level possible with the highest level of confidence. It took each botanist about 4 min of time to classify forages consumed by caribou in a one 9‐s video. Volunteer observers and botanists were required to review protocols and complete evaluations using training videos where we then could calibrate responses prior to starting data collection. Observers could also flag ambiguous videos for expert review. Random subsampling and data quality assurance and control procedures were developed and included for consistency.

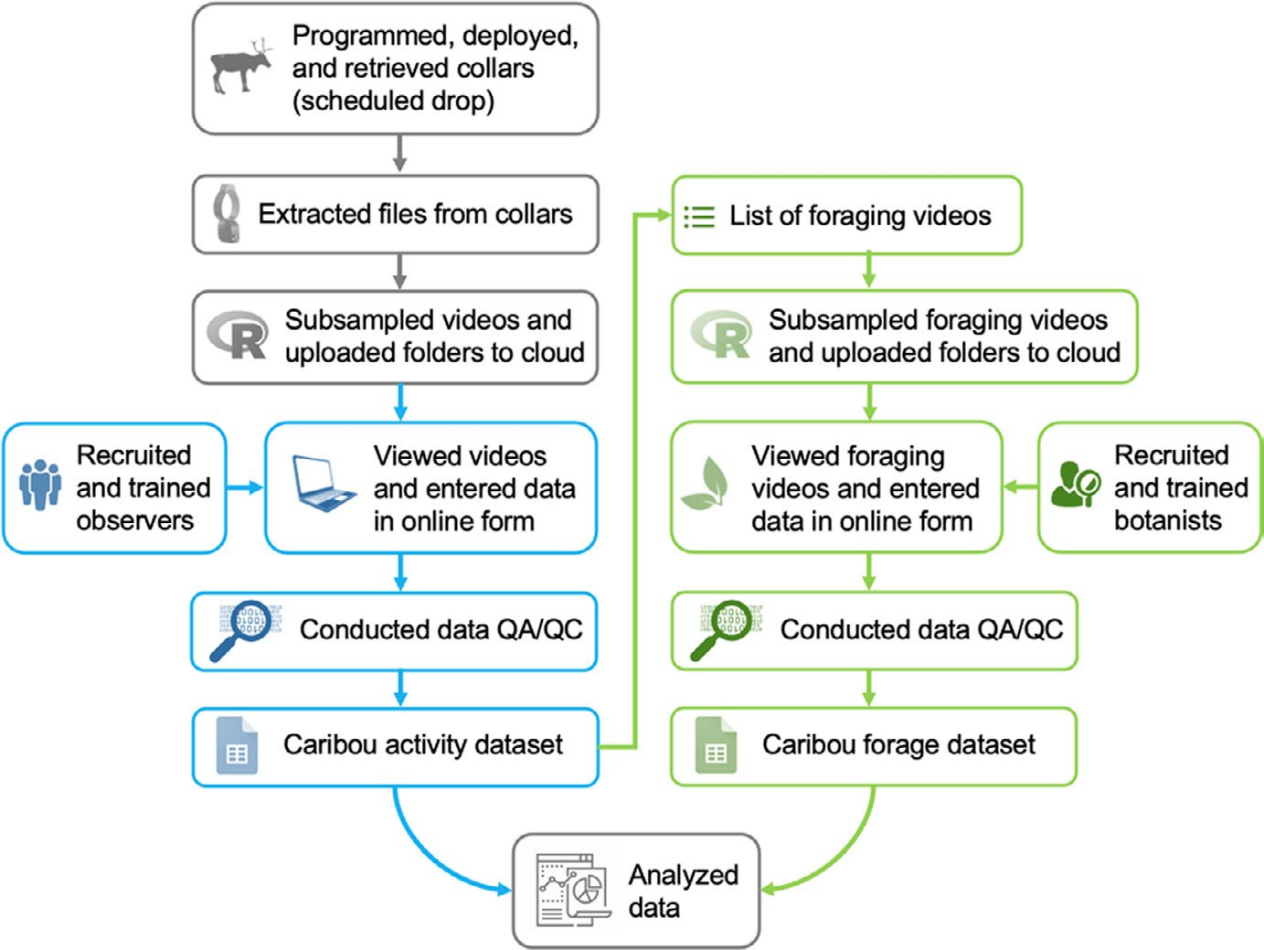

## 2 MATERIALS AND METHODS

### 2.1 Study area

**FIGURE 1:**
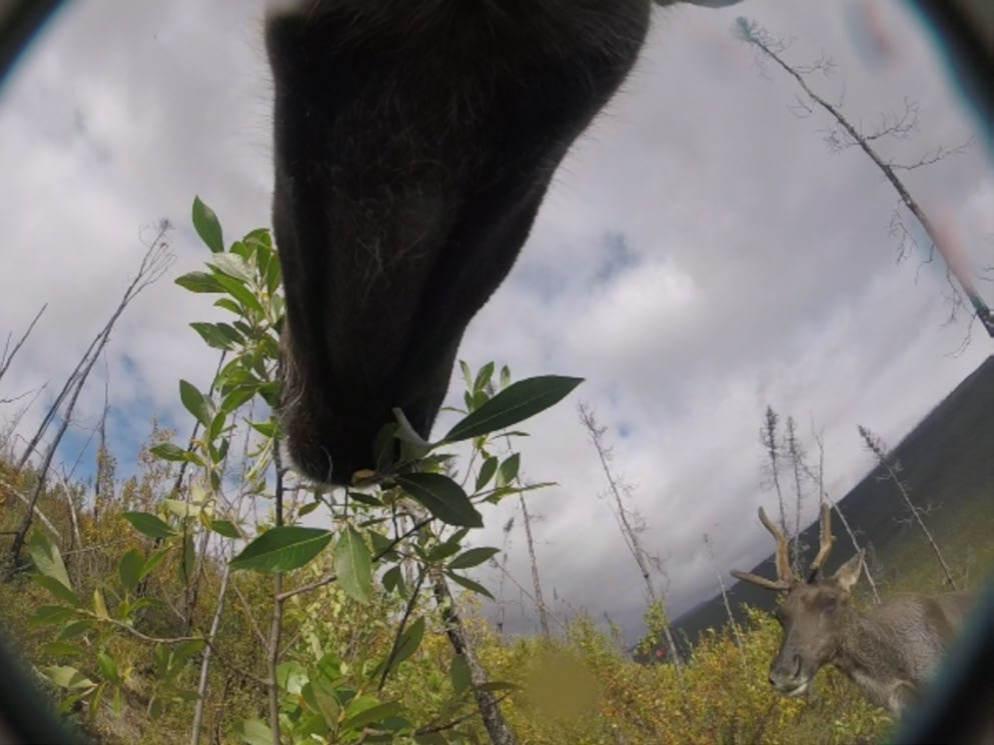
A female caribou of the Fortymile Caribou Herd (*Rangifer tarandus granti*) strips and consumes leaves from a *Salix pulchra* shrub. We classified behavioral and foraging activities for caribou during summer as observed from 9‐s videos recorded from GPS video‐camera collars across Alaska, USA and Yukon, Canada (2018 and 2019)

**FIGURE 2:**
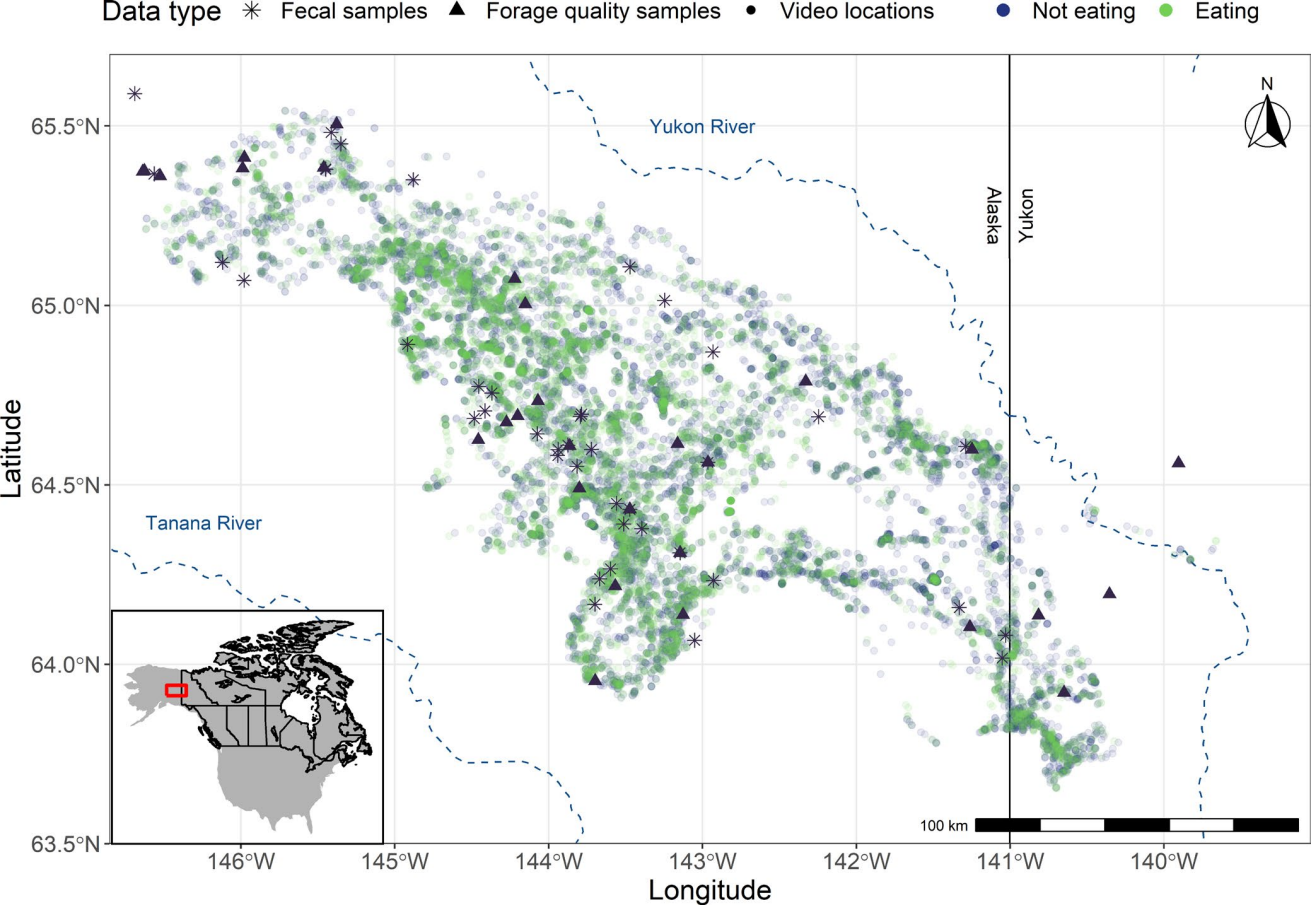
Study area for female caribou of the Fortymile Caribou Herd (*Rangifer tarandus granti*) across central interior Alaska, USA and North‐central Yukon, Canada. Caribou were outfitted with animal‐borne GPS video‐camera collars (*n* = 30) over two summers (2018 and 2019). Citizen scientist volunteers classified videos into categories based on caribou behavior (*n* = 18,134 videos). Circles represent the spatial distribution of all classified video locations for caribou, and colors highlight behaviors classified as eating (green; *n* = 5,549) and not eating (purple; ruminating, travelling, stationary awake, napping or others)

The Fortymile Caribou Herd is a migratory population of caribou spanning a 105,200 km^2^ region across east‐central Alaska and north‐central Yukon (Canada; Figure 1). The Fortymile Caribou Herd has increased from around 52,000 in 2010 to >84,000 in 2017 (Figure 2; Boertje et al., 2017; Harvest Management Coalition, 2019), spurning concerns regarding deteriorating summer range conditions and nutritional limitation. The bioclimate is characterized by long, cold winters (minimum temperatures = −50°C) and short, warm summers (maximum temperatures = 37°C). Precipitation is light in summer (mean 300–600 mm) and moderate in winter (average 1.5 m as snow), and fires are frequent and widespread (Jorgensen & Meidinger, 2015). Vegetation types include subalpine spruce (*Picea* spp.) forests, deciduous forests, shrubland and herbaceous tundra (Wang et al., 2020). Treeless herbaceous and tussock alpine tundra dominate landscapes above 800 m that also provide important habitats for calving, post‐calving and late summer aggregations that help minimize insect harassment (Boertje et al., 2017).

### 2.2 Ethics statement

All animal captures were conducted by the Alaska Department of Fish and Game and approved in accordance with animal welfare standards (IACUC permit numbers through ADFG 0002‐2018 and 0002‐2019).

### 2.3 GPS video‐camera collars

During March and April of 2018 and 2019, a total of 30 adult female (2018 = 15, 2019 = 15) caribou were captured from a helicopter with a netgun (*n* = 18) or tranquilizer dart (*n* = 12; Carfentanil/Xylazine). Caribou were then fitted with a GPS‐Iridium collar integrated with a camera and pre‐programmed with a drop‐off mechanism programmed to release on September 10 each study year (VERTEX Plus Iridium V 3.0, Vectronic Aerospace GmbH, Germany).

Video collars were programmed to record videos during daylight hours (14–18 h/day). For all programming periods from May to September, collars recorded a 9‐s video and GPS location every 20 min during daylight hours (Appendix A). Videos were processed using a two‐phased approach. First, trained volunteers classified a random subset of videos to classify caribou behavior (see Box 1, in blue; Appendix A). Second, videos classified as “eating” were viewed by five botanists with subarctic classification experience to identify species of forage(s) consumed by caribou (Box 1, in green).

### 2.4 Caribou behavior

We classified caribou behavior from videos into states of eating, ruminating, travelling, stationary awake, napping and others. We explored differences in behavior between/across (1) individuals, (2) years and (3) months, and contrasted frequencies of videos classified into different behaviors using one‐way Chi‐square goodness‐of‐fit contingency tests (GOF; Sokal & Rohlf, 1995). We used one‐way tests as an initial simple analysis step to explore temporal and individual behavioral differences. We could not consider two‐ or three‐way tests (e.g., to account for year/month by individual differences) because we radiocollared different individuals between years. We acknowledge that such one‐way tests likely commit type I error but used these as an initial exploratory step to focus subsequent statistical analyses of the main behavioral axis, changes in foraging. We also quantified insect avoidance behaviors observed in videos (e.g., shook head, scratched, sought snow patch, kept muzzle to ground and huddled; Morschel & Klein, 1997; Witter, Johnson, Croft, Gunn, & Gillingham, 2012; Witter, Johnson, Croft, Gunn, & Poirier, 2012; see Appendix A).

To test for the effects of insect harassment on eating in videos, we used generalized mixed‐effect models (GLMER, lme4 package in R, R Core Team, 2020) with a binomial (logit) link (Bates et al., 2015). We tested for the effects of the presence of insect avoidance behaviors (binary) on eating (binary) by female caribou in each video. Eating and insect avoidance behaviors were treated as events, suitable for analysis of frequencies (Altmann, 1974). We considered a random intercept to test for variation in eating between individuals and, in so doing, treated the individual as the sampling unit for all video‐based GLMER analyses. We also tested for a random coefficient for individual caribou and their individual variable responses to insect harassment (random coefficient; Appendix B Table B2). Model selection was performed using BIC selection criterion (Brewer et al., 2016).

### 2.5 Diet composition using video collars

Botanists experienced in arctic plant classification identified forages consumed to the most refined taxonomic level possible while still maintaining a high level of confidence (e.g., *Salix* spp., *Salix pulchra*; Box 1). If forage identification was uncertain, then videos were reviewed for a second opinion to confirm forage(s) selected by caribou. We calculated diet for each taxonomic unit as binary (yes, no) for each video and estimated diet as the percentage of videos classified as “eating” for that taxonomic unit. Diet composition estimated from video cameras is expressed as absolute percentages, as the sum of the percentages from different forage types could exceed 100% (because more than one forage type could be consumed in a one 9‐s video).

### 2.6 Diet composition using microhistological analysis

We collected fecal samples across the summer range of the Fortymile Caribou Herd over a 7‐year period (2011–2018), as a second estimate of summer caribou diet. Fecal pellet collection was targeted in areas with locations from GPS radiocollared females. Such locations represented an unknown mix of ages and sexes, though predominantly females based on GPS collar locations. Fecal samples were obtained from up to 25 distinct pellet groups and combined into a composite sample for each collection site. Unlike the video diet analysis, the composite fecal sample was the sampling unit during microhistological analyses (*sensu* Hebblewhite et al., 2008). Samples were stored frozen and later shipped to the Wildlife Habitat and Nutrition Laboratory at Washington State University for diet analysis. Diet composition was estimated by histological analysis of plant fragments with identification occurring at the coarse (B100; identifying species with >5% occurrence) or fine (A150; identifying all species occurrences ≥ trace levels) scale because of budget fluctuation. We removed rare forage types (those making up <4.0% of composite sample) and reported the mean diet of major plant classes (genera, species) averaged across each month from 2011 to 2018. Diet composition estimated from fecal microhistological analysis is expressed as a relative percentage, as the sum of percentages from different forage types sum to 100%.

### 2.7 Comparing methods to estimate summer diets

#### 2.7.1 Taxonomic resolution

We tested the taxonomic resolution between diet composition estimates from video collars and microhistology. We focused on the seven forage functional types (FFT) that occurred across both video collar and fecal data sets: *Equisetum* spp., forb, graminoid, lichen, moss, mushroom and shrub. We excluded forage types estimated as unknown or represented broader classes (e.g., ground‐cover vegetation).

#### 2.7.2 Correcting fecal diet samples for digestibility

We measured apparent dry‐matter digestibility (DMD in %; Van Soest, 1982) for plants consumed by caribou to correct fecal samples for digestibility to facilitate comparison to video‐collar‐derived diet estimates. We collected plant samples across the summer range of the Fortymile Caribou Herd from May to September for two summers concurrent with video collar deployment (2018 and 2019; Figure 2). Plant samples were air dried, weighed and stored in paper bags. Samples were dried in a ventilated drying oven at 65°C for 48 h (to a constant weight) and analyzed for detergent fibers (Van Soest, 1982), crude protein and tannin concentrations with bovine serum albumin (BSA; Martin & Martin, 1982) at the Wildlife Habitat and Nutrition Laboratory (Pullman, Washington, USA). We calculated DMD and adjusted for tannin content using Equations (1) and (2) of Hanley et al. (1992). For those forage functional types not assessed for forage quality by our team, we used DMD values estimated for the nearby Denali Caribou Herd (Boertje, 1990).

#### 2.7.3 Correlation of methods

Because we observed no differences in the frequency of eating between years from our initial Chi‐square tests, we lumped all years together. To test for similarities in diet composition estimated from video collar and fecal samples, we first applied the correction factor to our microhistological results to account for digestibility using our values for DMD (see details in Appendix B Table B4). We then compared, for each month, the six FFTs in the diet shared by video collar and fecal estimates; thus, we dropped the FFT for mushrooms because of their absence in microhistological analysis. We included May–August, as fecal samples were not collected in September. Forages that made up small portions (<1%) of the diet, as estimated by microhistological analysis, were removed. Next, we compared proportions of forage functional types between methods using Chi‐square tests. Finally, because of their large prevalence in the summer diet (see Section 3), we tested for correlations between the proportions of lichen and shrubs estimated by video collars and fecal pellets.

## 3 RESULTS

### 3.1 GPS video‐camera collars

Videos recorded data from 30 female caribou between May 10 and Sept 11 during 2018 and 2019. Two females died (May 12, 2018 and July 7, 2019), and two collars malfunctioned and stopped recording videos (final videos recorded on July 2, 2019 and August 7, 2019). We used data from collars prior to death or failure. We obtained a total of 176,150 videos over two summers (2018 and 2019). We viewed and collected behavioral data from 45.34 h of video footage that consisted of 18,134 videos (2018 = 12,484; 2019 = 5,650). We worked with 91 volunteer observers who qualified through the evaluation process and logged approximately 604 h of effort to classify the 18,134 videos. Video quality was subjectively classified as fair, good or excellent in 91% of video clips, poor in 8% and extremely obstructed in 1%. In most of the “extremely obstructed” videos, data could reliably be collected; most obstructions (71%) occurred as caribou foraged on ground‐level vegetation, neck or jaw fur obstructing the view, or as caribou napped (11%).

### 3.2 Caribou behavior

**FIGURE 3:**
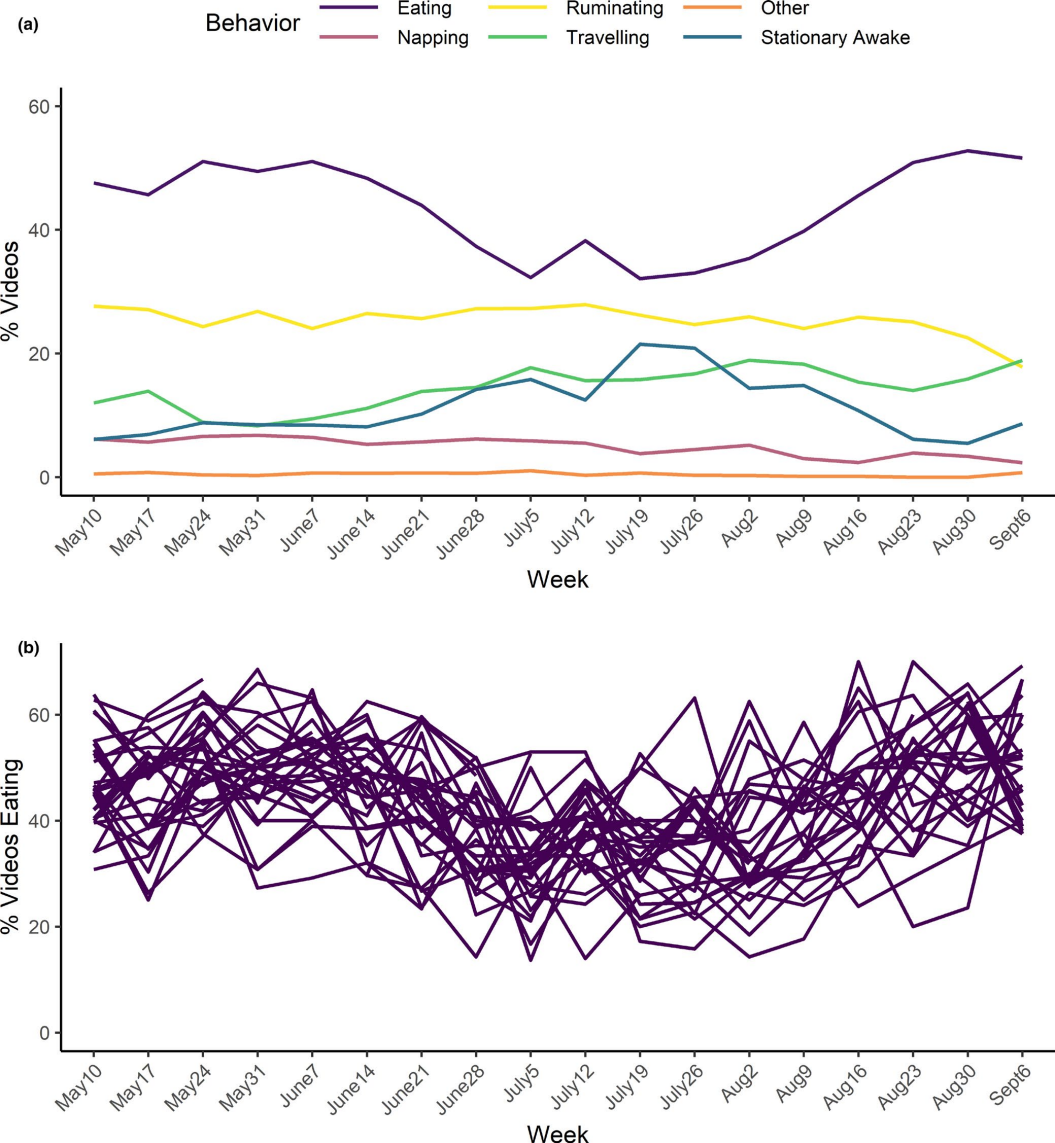
The proportion of videos (%) where caribou were observed (a) in different behavioral activities and (b) eating for each individual caribou throughout the summer season. We monitored female caribou (*n* = 30) of the Fortymile Caribou Herd (*Rangifer tarandus granti*), Alaska, USA and the Yukon, Canada during summer daylight hours, May–September 2018–2019

Caribou partitioned their behavioral activities into eating (mean = 43.5%), ruminating (25.6%), travelling (14.0%), being stationary awake (11.3%), napping (5.1%) and others (0.5%; e.g., drinking, licking soil for minerals and wading; Figure 3a). Summer behavioral activities for caribou did not differ between years (*χ* ^2^ = 7.55, *df* = 5, *p* = .18); therefore, we lumped data between years. Behavior did vary across months (*χ* ^2^ = 512.9, *df* = 20, *p* < .001) and individual females (*χ* ^2^ = 444.2, *df* = 145, *p* < .001; Figure 3b). We acknowledge the lack of independence of individual caribou in the Chi‐square GOF tests casts doubt on the strength of the *p*‐values. Nevertheless, they helped confirm that the main state driving changes in behavioral activity of caribou seemed to be the reduction in eating in July and not differences between individuals or years (Table 2, Figure 3). Subsequently, we thus focused on exploring foraging.

**FIGURE 4:**
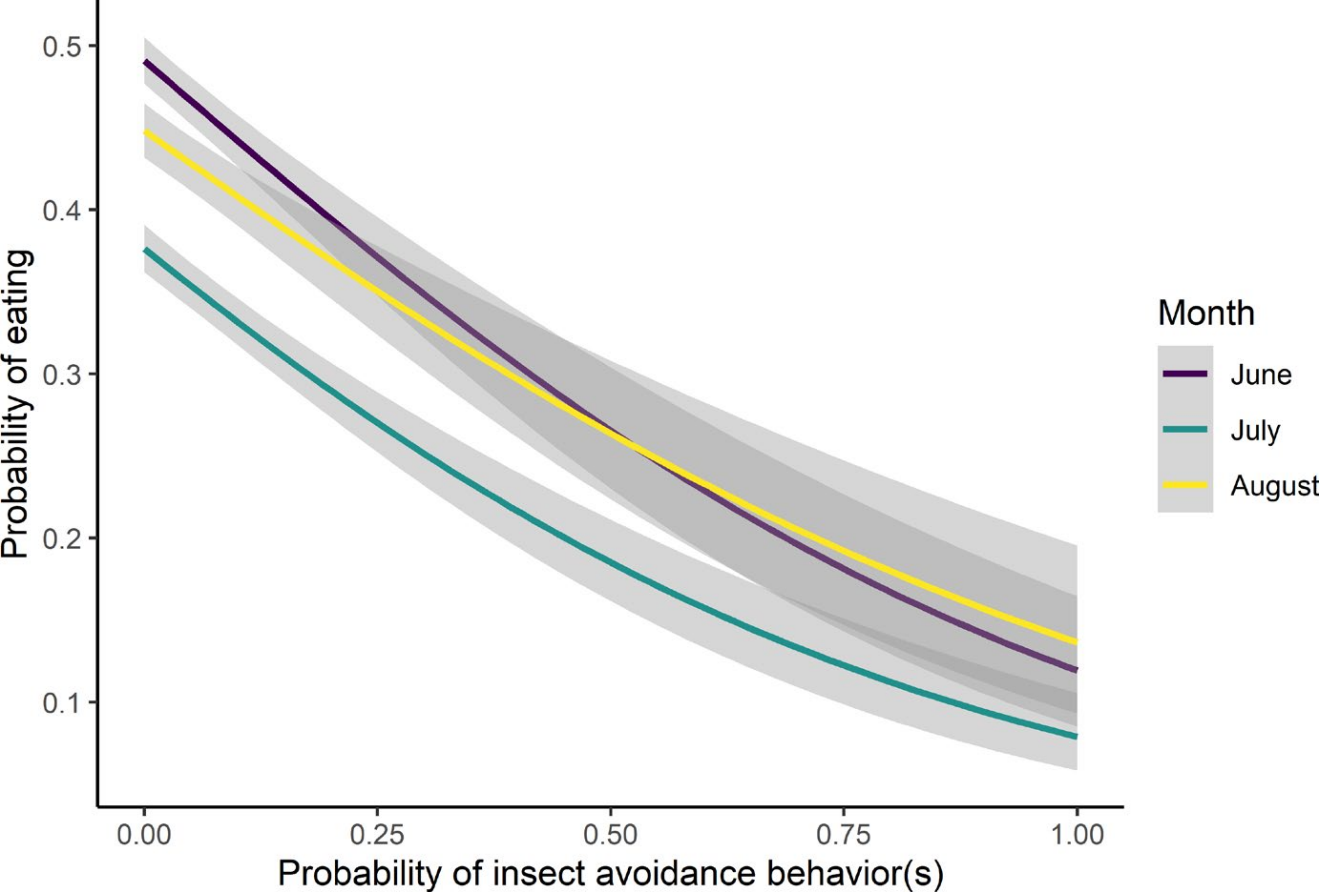
The relationship between the probability of eating and insect avoidance behaviors observed within 9‐s videos for female caribou of the Fortymile Caribou Herd (*n* = 30; *Rangifer tarandus granti*), Alaska USA and Yukon, Canada, 2018 and 2019. As the probability of insect avoidance behaviors increased, the probability of eating by caribou decreased. The probability caribou reduced eating while displaying insect avoidance behaviors varied across months

**FIGURE B1:**
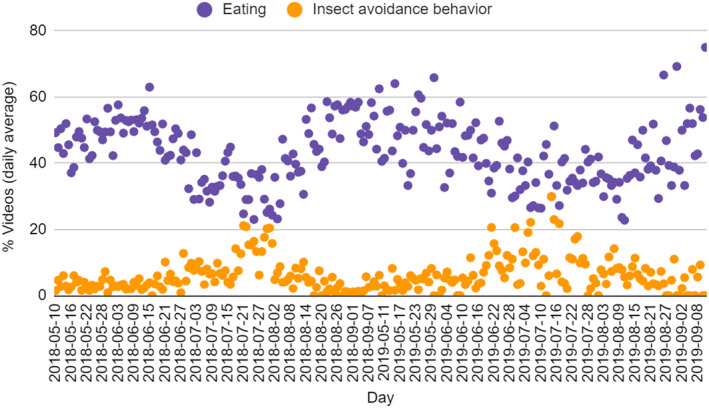
The proportion of videos (%) where caribou were observed eating (purple) and/or displaying insect avoidance behaviors (orange). The proportion of videos (%) was calculated as daily averages but summarized by week for improved visualization. Data were collected from GPS video‐camera collars during summers 2018 and 2019. Although the temporal scale looks continues, years transition in center of figure (“2018‐09‐07” to “2019‐05‐11”)

Insect avoidance behaviors increased through July and were associated with reductions in the frequency of eating (Figure 4; Appendix B Figure B1). Our most parsimonious model (Table 1) showed a strong negative effect of insect harassment on the probability of eating for caribou (*β* = −2.02, *p* < .001; Table 2). The standard deviation (SD = 0.1) of the random effect suggests responses among individual females did not vary strongly. The second ranked model (Table 1) was the same as the top model without a random effect for individual. These results collectively support our Chi‐square analyses above showing minimal individual‐level variation in behavior and eating (Figure 3b), and the consistency in the tradeoff between insect avoidance behaviors and eating. These conclusions are also supported by the tradeoff at weekly eating scales (see Appendix B Figure B1).

**TABLE 1:** The five most parsimonious models, based on ∆BIC values, from a set of candidate binomial generalized linear models of the effects of insect harassment on the frequency of foraging events observed in videos throughout the summer months for caribou of the Fortymile Caribou Herd (*Rangifer tarandus granti*), Alaska, USA and Yukon, Canada, 2018 and 2019

Model	Model name	BIC_w_	BIC	ΔBIC	*df*
1	Insects + MonthF + (1 | CamID_Yr)	24,041	0	0	7
2	Insects + Month	24,044	2.7	2.7	6
3	Insects + Year + Month	24,049	8.4	5.7	7
4	Insects + MonthF + YearB + Insects * YearB + (1 | CamID_Yr)	24,051	10.1	1.7	9
5	Insects + MonthF + Insects * MonthF + (1 | CamID_Yr)	24,061	20	9.9	11

Note

Random effect for individual caribou (1 | Individual).

**TABLE 2:** Coefficient table from the most parsimonious logistic regression model explaining the probabilities of caribou (*Rangifer tarandus granti*) eating that included fixed effects for insect avoidance behaviors and month and a random effect for individual caribou of the Fortymile Caribou Herd, Alaska, USA and Yukon, Canada, 2018 and 2019

Fixed effects	Estimates (*β*)	SE	Pr(>|*z*|)	Probability of eating, without insect avoidance behavior (%, predicted GLMER)	Probability of eating, with insect avoidance behaviors (%, predicted GLMER)	Frequency of eating at the monthly scale (%, observed from videos)	Frequency of insect avoidance behaviors at the monthly scale (%, observed from videos)
Intercept (May)	−0.04	0.04	0.33	49.0	11.3	48.0	3.7
Insects	−2.02	0.11	<0.001	–	–		–
June	−0.01	0.04	0.85	48.9	11.3	47.2	5.2
July	−0.47	0.05	<0.001	37.6	17.4	34.5	10.5
August	−0.17	0.05	0.001	44.9	9.8	43.3	4.9
September	0.14	0.07	0.04	52.6	12.9	51.5	2.7
Average				45.5	10.5	44.9	5.4

Note

Included are the model predictions for the amounts of instantaneous (in 9‐s videos) probabilities for females eating (%) with and without insect avoidance behaviors. Also included are comparisons to the frequencies of eating and insect avoidance behaviors (%) from counts of the raw video footage averaged over the month.

### 3.3 Diet composition using video collars

**FIGURE 5:**
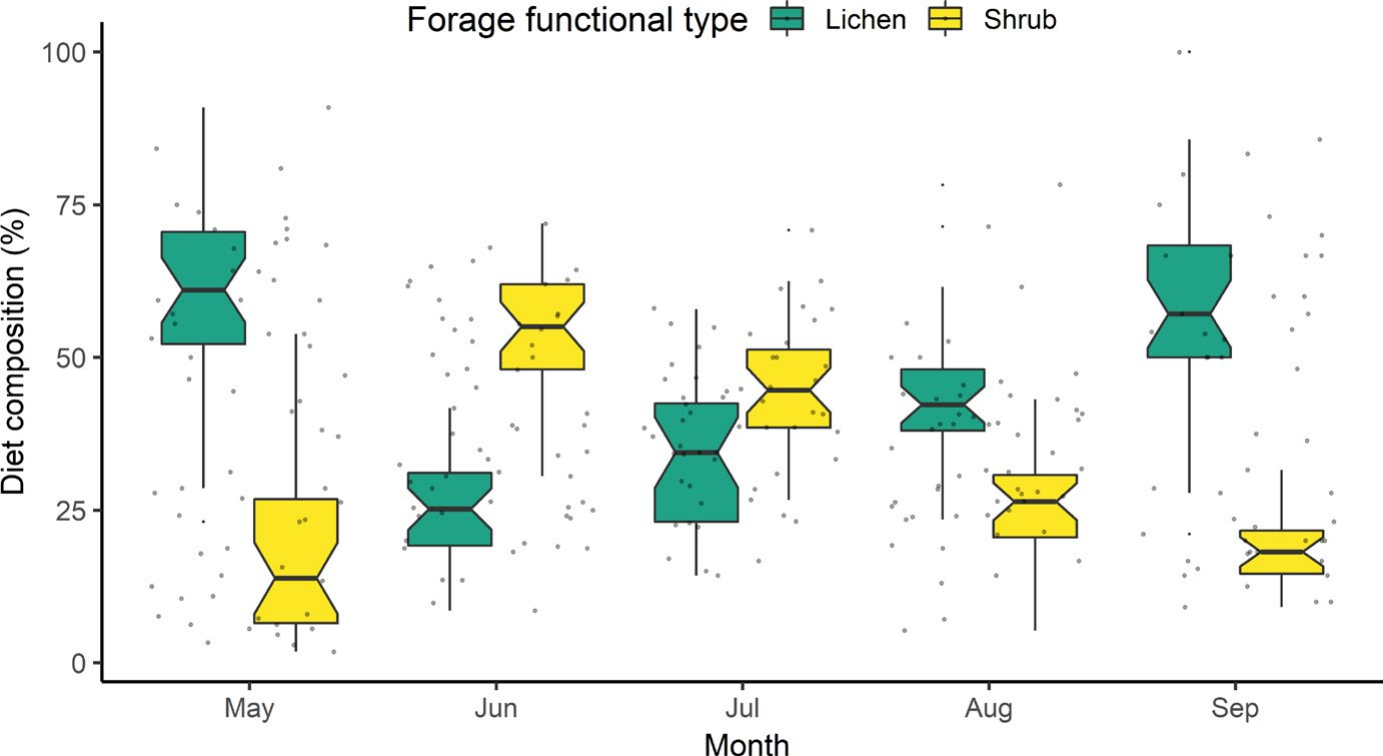
Notched boxplots quantify the proportion of lichen and shrub in the summer diets of female caribou (*n* = 30) of the Fortymile Caribou Herd (*Rangifer tarandus granti*). We identified forages consumed in 5,549 videos collected from GPS video‐camera collars during daylight hours (summers 2018 and 2019). Caribou diets estimated from video collars were composed primarily of lichens during the early and late summer season (May and September), trading off for shrubs in June and July. Boxes represent the interquartile range (IQR; 25%–75%); whiskers include 99.3% of data if normally distributed; lines represent the median values; and notches within boxes are the confidence interval around the median value

Five botanists expended 370 h of classification effort to collect diet data from 14 h of videos (*n* = 5,549; Appendix B Figure B4) and identified 7,529 foraging items. Botanists classified video quality as fair, good or excellent in 79%, poor in 14% and extremely obstructed in 7% of foraging videos. Forages were identified to species (mean = 32% of items), genus (32%), family (3%), forage functional type (15%), likely lichen (9%), unknown ground‐level vegetation (9%) or unidentifiable (<0.1%; Appendix B Table B4). The summer diet was classified into nine forage functional types: *Equisetum* spp. (summer mean = 0.1%), forbs (6.4%), graminoids (7.0%), ground‐level vegetation (8.7%), lichen (39.4%), moss (0.4%), mushroom (1.7%), shrubs (36.7%) and unknown forages (0.4%; Figure 5 and Appendix B Figure B5). Shrubs included *Salix* spp. (not identified to species; 16% of foraging clips), *Salix pulchra* (8%) and *Betula nana*/*glandulosa* (13%; Appendix B Figure B5). Dominant lichens were identified as belonging to the *Cladina*/*Cladonia* genera (18% of foraging videos; Appendix B Figure B5). Diet estimates from video collars highlight the tradeoff between lichen and shrubs in the diet, with shrubs dominating the diet in June and July (Figure 5).

### 3.4 Diet composition using microhistological analysis

**FIGURE 6:**
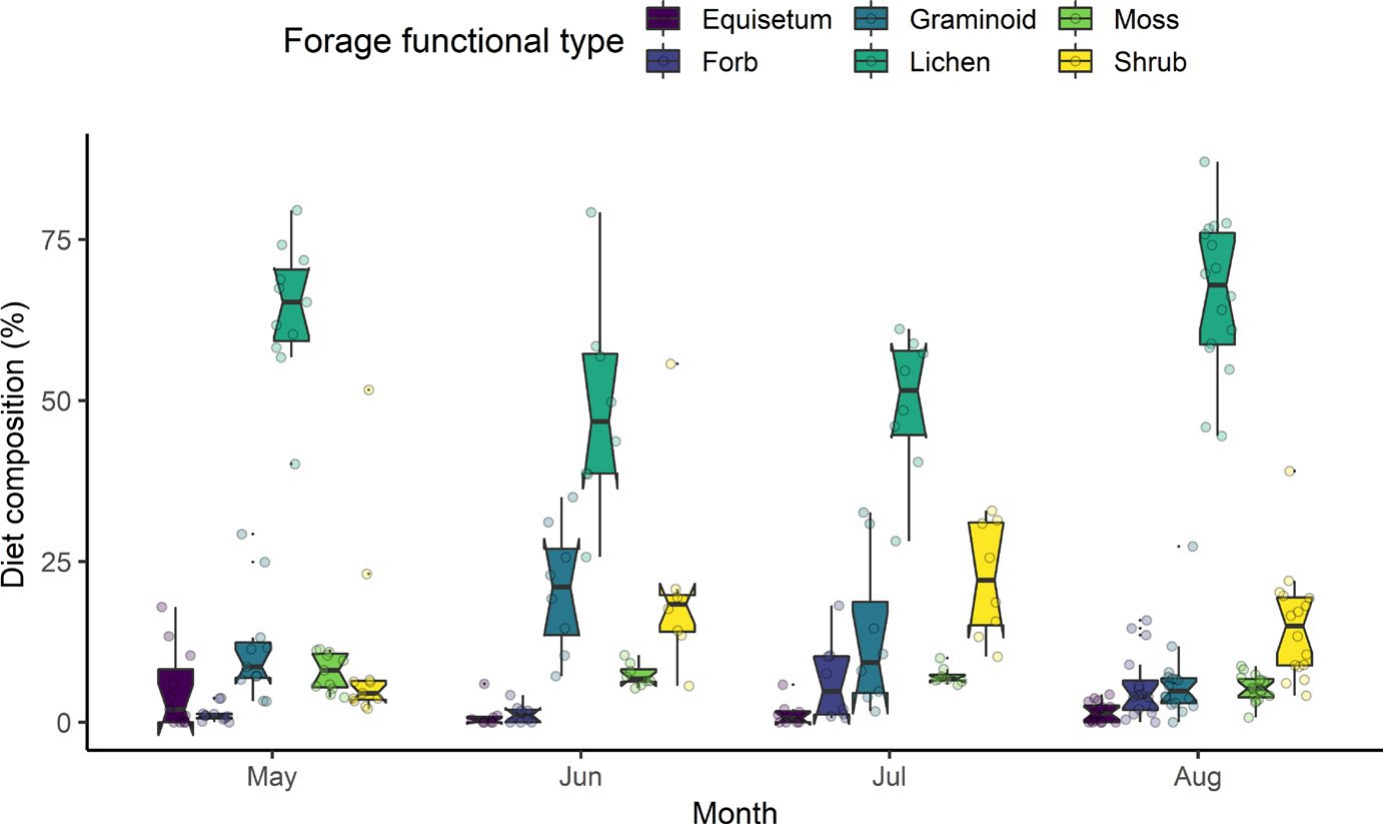
Notched boxplots represent the summer diets of female caribou of the Fortymile Caribou Herd (*Rangifer tarandus granti*) based on microhistological analysis (digestibility corrected). Raw diet data were classified across forage functional types, and composite fecal samples were collected over eight summers (*n* = 43; 2011–2018). Lichens constituted the highest proportions (median) in summer diets as per microhistological analysis. Boxes represent the interquartile range (IQR; 25%–75%); whiskers include 99.3% of data if normally distributed; lines represent the median values; and notches within boxes are the confidence interval around the median value

**FIGURE 7:**
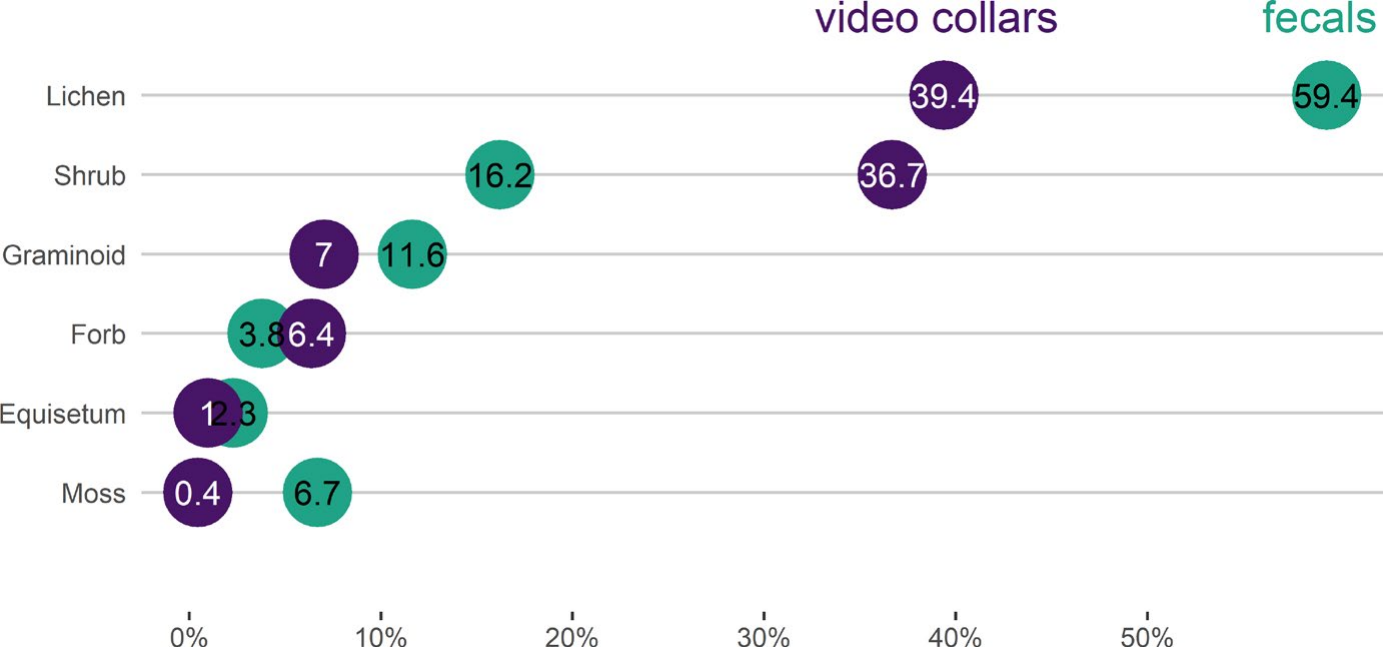
The mean proportions of six forage functional types (lichen, shrub, graminoid, forb, *Equisetum* spp. and moss) estimated in the summer diets of caribou of the Fortymile Caribou Herd Alaska, USA and Yukon, Canada, 2011–2019. Diet composition was estimated as the mean proportion for the six forage functional types found in both methods for individual caribou (sampling unit for video collars = “video collars”) and composite fecal sample (sampling unit for microhistological analysis = “fecals”). Diet composition estimates from video collars are expressed as absolute percentages (purple circles), and estimates from microhistological analysis are expressed as relative percentages (green circles)

We analyzed 43 composite fecal samples and adjusted microhistological results for digestibility. We classified forages into six forage functional types: *Equisetum* spp. (mean proportion in diet 2.3%), forbs (3.8%), graminoids (11.6%), lichen (59.4%), moss (6.7%) and shrubs (16.2%; Figures 6 and 7). Dominant shrubs included *Salix* spp. leaves and stems (not identified to species; mean proportion in diet 11.6%). Dominant lichens belonged to the *Cladina*/*Cladonia* genera (38.4%). Lichen dominated the diet across all months (Figures 6 and 7; Appendix B Figure B7).

### 3.5 Comparing methods to estimate summer diets

#### 3.5.1 Taxonomic resolution

We identified 63 species in 70 genera in 33 families of summer forages consumed by caribou using video collars (Appendix B Figure B9). Microhistological analysis identified plants to 12 species in 24 genera in six families using plant fragments found in fecal pellet samples.

#### 3.5.2 Correcting fecal diet samples for digestibility

We measured apparent dry matter digestibility (% DMD) for 167 plant samples across four forage functional types: shrubs (58.2%, *n* = 85), lichen (75.1%, *n* = 37), graminoids (72.9%, *n* = 37) and forbs (77.2%, *n* = 8; Appendix B Table B4). The concentration of tannins (mg BSA/mg forage) was calculated for 118 caribou forage samples. We then adjusted DMD for tannin precipitate, as tannins cause reductions in forage digestibility for ruminants. We considered *Equisetum* spp. highly digestible and used our DMD value for forbs (77.2%; *sensu* Boertje, 1990). For mosses, we used DMD values determined by Boertje (1990; 7%), as mosses have been shown to have poor digestibility (Ihl & Barboza, 2005). Our DMD values were highly correlated to Boertje's (1990), which allowed us to use their values with accuracy when needed (Appendix B Figure B8). Our shrub samples included some woody stems and therefore likely underestimated shrub digestibility and the resulting proportion of shrub in the corrected diet estimates.

#### 3.5.3 Correlation of methods

We found a positive correlation between the proportions of forage functional types estimated across months (*r* = 0.79, *p* < .01; Appendix B Figure B10) from video collar and digestibility‐adjusted microhistological methods (Figure 7). The relationship between summer diet estimates was marginally statistically significant (*r* = 0.79, *p* = .06). Diet estimates for monthly lichen (*r* = 0.81, *p* = .18) were not correlated between the video collar and microhistological methods; however, estimates for monthly shrub (*r* = 0.93, *p* = .07) were marginally statistically significant.

## 4 DISCUSSION

Animal‐borne video collars provided a powerful new tool to remotely assess behavioral and foraging patterns for large herbivores across remote regions. This tool allowed us to identify behavioral and nutritional tradeoffs that were previously difficult to detect with field observations and/or fecal plant fragment analysis. Behavioral activities for caribou varied strongly across the summer and were strongly driven by insect avoidance behaviors. Using video collars, we identified (1) higher dietary diversity by discerning forage types at finer taxonomic levels than fecal sampling and (2) a strong temporal tradeoff in the consumption of lichen and shrubs. Our work demonstrates video collars are useful, especially in remote regions like the arctic, to document behavior and diet.

We found managing and classifying videos took significant amounts of effort (Mattern et al., 2018). Recruiting and retaining volunteers were time intensive, and only 30% expressing interest completed the training to become observers. We incentivized student engagement with undergraduate independent research credits. Training volunteers, using data entry forms and evaluation processes, provided consistency in data collection. Out of 91 volunteer observers that completed training and collected data, few (*n* = 14) classified >300 videos. Similar to Thompson et al. (2015), hiring arctic plant experts to classify foraging videos provided the necessary skills for diet classification. Regardless, classification of videos took >hundreds of hours. Although we see the future of video classification as an automated process, it will be difficult to automate accurate diet classification from videos, and researchers should be prepared to allocate resources to processing diet data.

### 4.1 Caribou behavior

Our work demonstrates video collars can quantify behavioral activities across a variety of temporal scales: daily (e.g., Appendix B Figure B1), weekly, monthly, seasonally and yearly. Caribou spent an average of 45% of daylight hours eating in summer (Table 2). This is similar to other migratory populations in Alaska (40%–60%; Maier & White, 1998), the Canadian arctic (55%; Witter, Johnson, Croft, Gunn, & Gillingham, 2012; Witter, Johnson, Croft, Gunn, & Poirier, 2012), Quebec (55%; Toupin et al., 1996) and wild reindeer in Norway (47%; Colman, 2003). Consistent with other studies (Russell et al., 1993; Thompson et al., 2015), we also found little variation of behavioral activities for caribou across years that strengthens our temporal inference. This consistency in eating behavior across individuals also provides support for population‐level inferences.

Our results are also consistent with the foraging ecology of large herbivores in summer. Because summer forages are more digestible, ungulates reduce gut retention and rumination time, and increase intake rates (Barboza et al., 2009; Van Soest, 1982). As a result, passage rates become the limiting factor in ungulate nutrition during summer. Caribou spent just 25% of their time ruminating in summer, similar to previous summer studies (Maier & White, 1998; Russell et al., 1993), but much lower than winter when rumination accounts 40%–50% of the activity budget (Russell et al., 1993). Video collars also documented the evident tradeoff between eating and other behaviors, like insect avoidance and movement, foundational to mechanistic ungulate foraging models (e.g., Hobbs et al., 2003; Spalinger & Hobbs, 1992).

### 4.2 Foraging behavior and insect harassment

**FIGURE B2:**
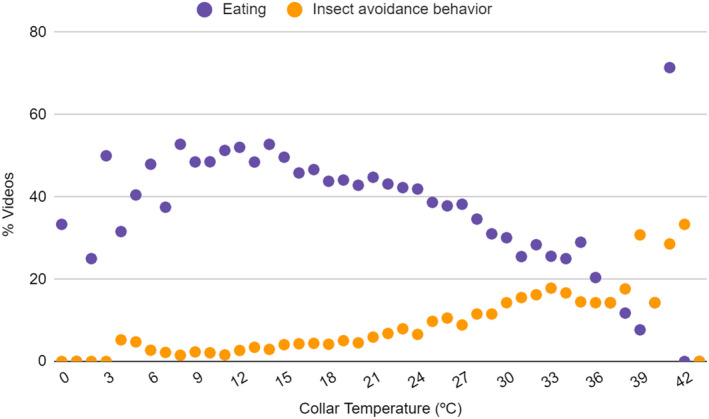
The proportion of videos (%) where caribou were observed eating (purple) and/or displaying insect harassment behaviors (orange) in relation to temperature (°C) as recorded by the GPS video‐camera collars. Data were recorded from 30 female caribou of the Fortymile Caribou Herd across Alaska, USA and Yukon, Canada over two summers (May–September; 2018 and 2019)

Our results show interior populations of migratory caribou reduce eating when exposed to insect harassment as predicted and based on other studies. Reductions in the probability of eating by caribou correlated strongly with increased probability of insect avoidance behaviors (Figure 4) and temperatures in July and were not correlated with the increase in shrub consumption (Appendix B Figure B2). Caribou reduced their frequency of eating from 48% in May to 34.5% in July (Figure 3, Table 2). These reductions in eating are similar to observations of coastal populations of migratory caribou. Caribou summering on the coastal plains of Alaska and the Yukon (Russell et al., 1993), as well as in alpine tundra (Morschel & Klein, 1997), reduced feeding time from 60% to 25% under insect harassment. In the Northwest Territories and Quebec, Canada, Witter, Johnson, Croft, Gunn, and Gillingham (2012), Witter, Johnson, Croft, Gunn, and Poirier (2012) and Toupin et al. (1996) found caribou fed only 30%–38% of the time in the presence of oestrid (e.g., bot fly) insect harassment. Similarly in Norway, semi‐domesticated migratory reindeer reduced their feeding to 23% under insect harassment (Colman et al., 2003). Although fewer studies have quantified foraging reductions for interior populations in Alaska (Boertje, 1985; Maier & White, 1998; Morschel & Klein, 1997), our work shows that interior caribou face similar costs of insect harassment as coastal populations.

Past studies in the arctic have shown mosquitoes (*Culicidae*) alter forage selection and induce behavioral responses by caribou (e.g, grouping and movement; Johnson et al., 2021; Joly et al., 2020; Witter, Johnson, Croft, Gunn, & Gillingham, 2012; Witter, Johnson, Croft, Gunn, & Poirier, 2012). The avoidance behaviors we frequently observed (e.g., muzzle to the ground, head shaking, stomping and scratching), however, suggest harassment by oestrids (*Oestridae*) and tabanids (*Tabanidae*). In addition, caribou collar temperature (an indicator of oestrid insect activity; Appendix B Figure B2) had a strong negative correlation with the frequency of eating. As temperatures rise due to climate change, insect activity is predicted to increase across the arctic (Koltz & Culler, 2021; Mörschel, 1999; Witter, Johnson, Croft, Gunn, & Gillingham, 2012; Witter, Johnson, Croft, Gunn, & Poirier, 2012), potentially further reducing summer foraging (Appendix B Figure B2).

**FIGURE B3:**
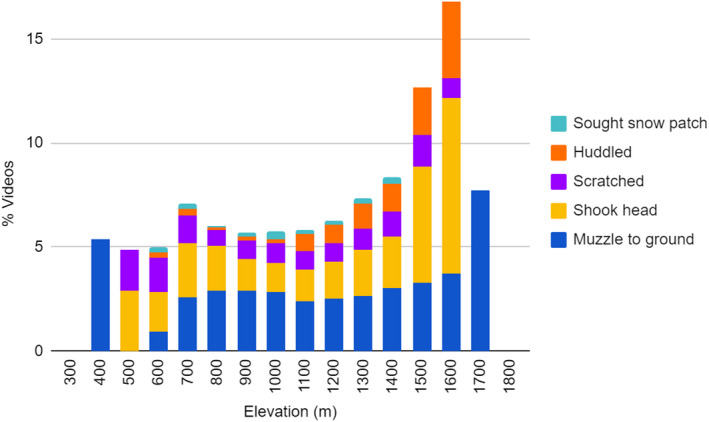
The proportion of videos (%) where caribou displayed insect avoidance behaviors (teal = sought snow patch, purple = scratched, gold = muzzle to the ground, orange = huddled and navy = shook head) in relation to elevation (m; rounded to nearest 100 m) as recorded by GPS video‐camera collars. Data were recorded from caribou (*n* = 30) of the Fortymile Caribou Herd across Alaska, USA and Yukon, Canada over two summers (May–September; 2018 and 2019)

**FIGURE B4:**
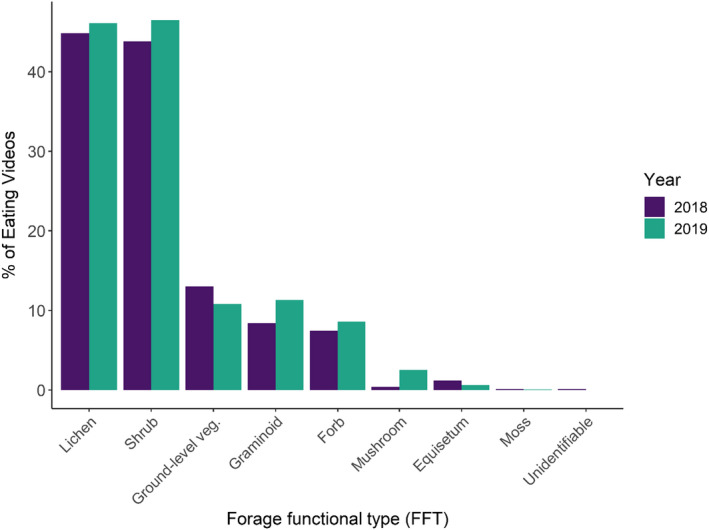
Annual diet estimates from GPS video‐camera collars for 30 female caribou of the Fortymile Caribou Herd. We identified forages from 5,560 videos (2018 = 4,500; 2019 = 1,060). Because of efforts to classify videos, we assessed behavior and eating patterns at 1,000 classified foraging videos in 2019. Because frequencies of behavior (% of videos) and eating (% eating videos by forage functional type) were similar between years, we terminated classification efforts of videos in 2019 to progress with analyses

**FIGURE B5:**
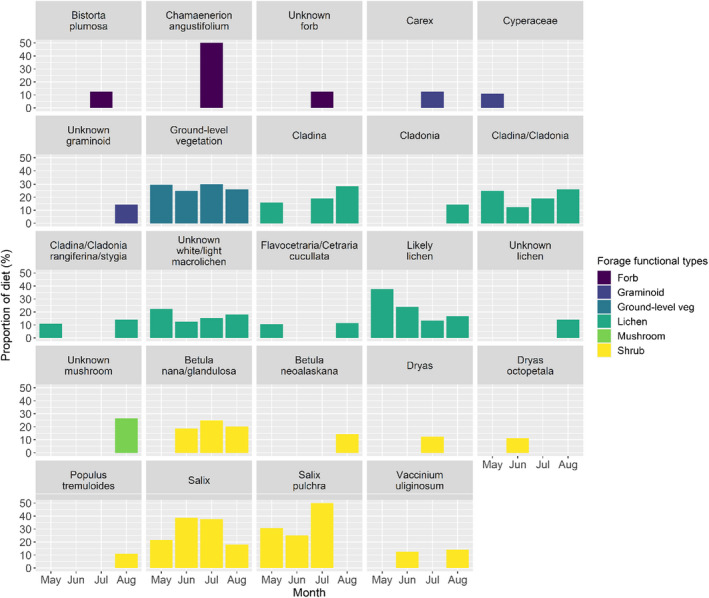
Summer diet composition to the most refined taxonomic level for caribou (*n* = 30) in the Fortymile Caribou Herd based on GPS video‐camera collars. Species included are those making up ≥10% of the summer diet each month

**FIGURE B7:**
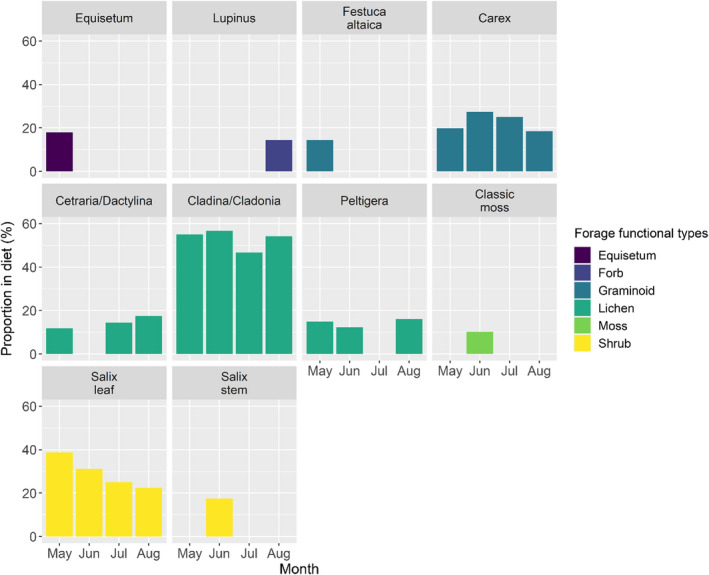
Summer diet composition to the most refined taxonomic level, corrected for digestibility, for caribou in the Fortymile Caribou Herd based on microhistological analysis (*n* = 43). Forage types included are those making up ≥10% of the total diet

**FIGURE B8:**
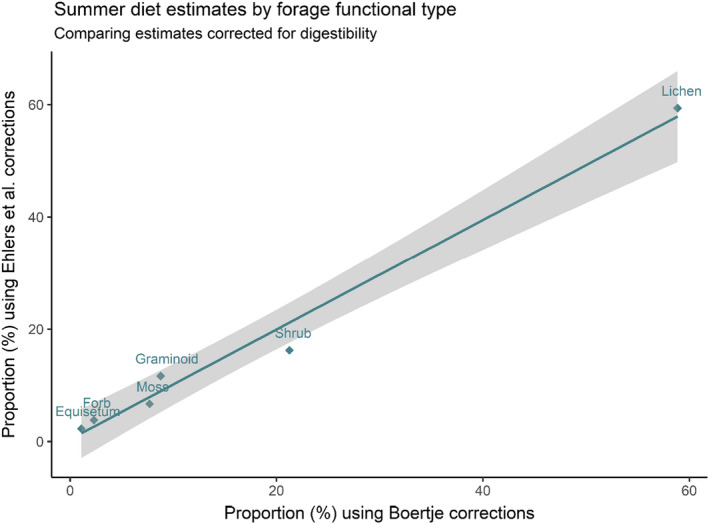
Testing correlations between the proportions of six forage functional types (FFT), corrected for digestibility, consumed by caribou of the Fortymile Caribou Herd across Alaska, USA and the Yukon, Canada. Correlations compare summer diets estimated using Ehlers et al. and Boertje’s (1990) DMD correction factors to account for digestibility in microhistological analysis (Table B4)

As eating decreased when insect avoidance behaviors increased, movement also increased similar to other studies (Figure 3a; Hagemoen & Reimers, 2002; Joly et al., 2020; Russell et al., 1993). For example, the Western Arctic Caribou Herd moved nearly twice as much during insect harassment periods (Joly et al., 2020). These increased movements can decrease foraging opportunities. Instead, caribou in mountainous areas travel from nutrient‐dense lower‐elevation habitats to high‐elevation, nutrient‐poor vegetation communities in alpine to seek relief from insects on wind‐blown ridgelines (Appendix B; Figure B3; Russell et al., 1993; Anderson et al., 2001).

The joint effects of reduced foraging and increased movement can lead to high energetic costs. Caribou may be unable to compensate or replenish energy reserves lost from reduced foraging (Colman et al., 2003) especially during summer, the critical time female ungulates improve body condition for lactation and year round nutrition (Cook et al., 2004, 2021; White et al., 2013). We studied the effects of insect harassment on females, but juveniles experience immediate and more severe consequences than adult females from increased stress, low weight gain and, in rare cases, death (Helle & Tarvainen, 1984; Weladji et al., 2003). In the future, researchers could pair accelerometers with foraging and insect data from videos to calculate the true energetic costs of extra movement across age and sex classes (Williams et al., 2014). Our estimates of tradeoffs between eating and insect avoidance behaviors could be also used in energetics models (e.g., White et al., 2014) to understand consequences of changes in insect harassment to populations.

There are several caveats to consider in analyzing complex behavioral responses across time, space and individuals. First, we acknowledge behavior is obviously an explicitly multivariate process, and our bivariate analyses of tradeoffs between insect avoidance behaviors and eating likely overlooked this multivariate process. However, we used random effects for each individual female caribou, with new individuals radiocollared each study year, to account for individual heterogeneity in foraging behavior (Gillies et al., 2006). Thus, we choose to account for the sample unit of individual animals in the GLMM with a random effect for individual instead. This demonstrated weak individual‐level variation, for example, a key finding of our study. It is also important to acknowledge the temporal sampling scale of our behavioral activity within 9‐s videos, a near‐instantaneous foraging scale (e.g., on average, we classified 4.8 videos/day/caribou for behaviors and 1.5 videos/day/caribou for identifying foraging items). This instantaneous scale likely overestimated the tradeoff between eating and insect avoidance behaviors at daily or longer foraging scales, following theory on upscaling foraging of ungulates (Fryxell, 1991; Spalinger & Hobbs, 1992). For example, in Table 2, the probability of eating while also being harassed by insects was 17.4% in July in 9‐s videos. But, averaged over 1 month, insects reduced the frequency of eating by 10.5% (Table 2, Appendix B Figure B1). However, the tradeoff between eating and insect avoidance behaviors was evidenced not only within 9‐s videos but also when looking at means across all temporal scales. And our estimates from instantaneous scales were similar to previous studies that demonstrated reductions in foraging activity from direct observations (e.g., Witter, Johnson, Croft, Gunn, & Gillingham, 2012; Witter, Johnson, Croft, Gunn, & Poirier, 2012).

Throughout the boreal forest, caribou and elk show similar responses to insects (Gates & Hudson, 1981; Raponi et al., 2018). Insect harassment is critical not only for caribou summering along the arctic coasts but also for interior subarctic populations in alpine tundra, as our results show, and for large herbivores around the world. Many components of herbivore ecology and evolution are driven by insect harassment, so much so that zebra (*Equus burchelli* or *E*. *quagga*) evolved stripes to confuse and prevent flies from landing and probing for blood (Caro et al., 2019). Global changes in environmental conditions may alter the distribution and abundance of parasitic insects in ways that reduce nutritional condition of large herbivores, especially in arctic regions (Joly et al., 2020). Future studies could similarly use video collars to investigate insect‐herbivore ecology.

### 4.3 Summer diets

**FIGURE B9:**
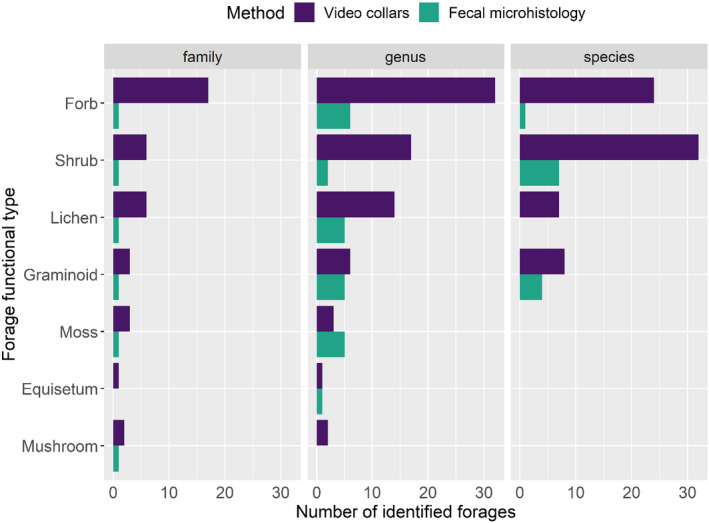
Total number of forages consumed by caribou across taxonomic levels for each of two methods used to assess the summer diet composition for the Fortymile Caribou Herd(*Rangifer tarandus granti*) across Alaska, USA and the Yukon, Canada. Forageswere classified to their forage functional type (FFT) from GPS video‐camera collars (purple = video collars) and fecal samples (green = fecal microhistological). Seven FFTs (*Equisetum* spp., forbs, graminoids, lichen, moss, mushroom, and shrubs) were included and available across methods for comparison

**FIGURE B10:**
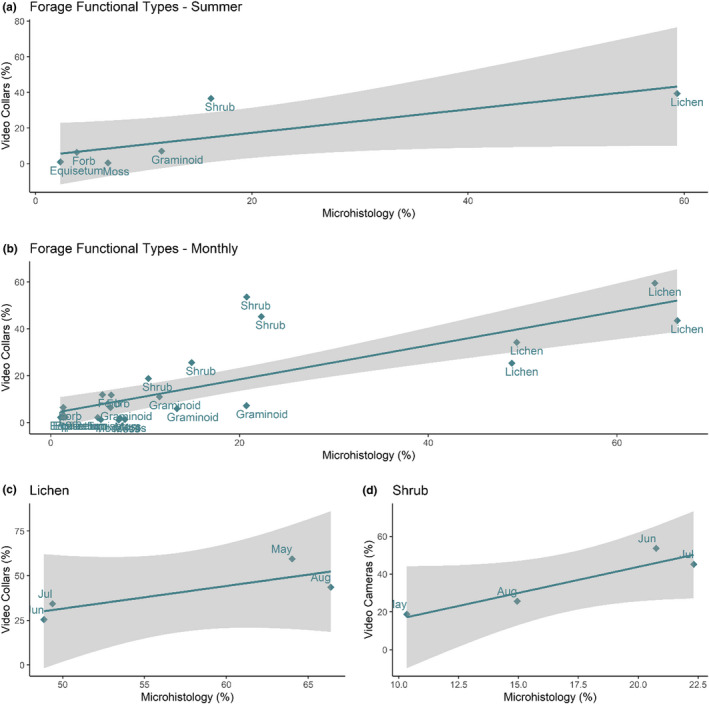
Testing correlations between two methods for estimating the diet composition for female caribou (*Rangifer tarandus granti*) using video collars and microhistology. Correlations were analyzed across six forage functional types (FFTs) common across both methods for (a) summer (b) each month and for (c) lichen and (d) shrubs due to their contributions to the summer diet of caribou

We found video collars provided greater taxonomic resolution of diet that correlated with traditional methods (Lavelle et al., 2015; Newmaster et al., 2013; Parrish et al., 2005). We identified >60 species from videos but only 12 species from fecal samples (Appendix B Figure B9). Some taxonomic groups were difficult to identify from cameras, like those we lumped into the “ground‐level vegetation” category. But it remained challenging to discern forages at levels finer than the forage functional type or genera level using microhistological analysis. Furthermore, the finer the taxonomic level, the greater the discrepancy between diet from video collars and microhistological analysis (Appendix B Figure B9). Newmaster et al. (2013) and Thompson et al. (2015) first used video collars to document seasonal diets of woodland caribou, noting some of these same discrepancies but did not account for digestibility when comparing fecal results to videos. Accordingly, Newmaster et al. (2013) found summer diets estimated from fecal samples to be <15% correlated with estimates from video cameras. After accounting for digestibility, our diet estimates were correlated between methods for all forage functional types estimated across months but not within lichen or shrub functional types. For lichen and shrubs, videos indicated a tradeoff of these two forage types (Figure 5), whereas microhistological analysis estimated lichen as the dominant food item consumed by caribou all summer (Figure 6). While videos are insightful, fecal samples likely misrepresent dietary composition due to higher digestibility levels of shrubs. Differences could also arise because of sex‐based diet differences (videos were only on females) or, more likely, spatial sampling bias of fecal pellet collection (see Figure 2). Despite costs of the collars and deployment, video collars provide large and systematic sample sizes of videos during daylight hours, extensive spatiotemporal coverage and strong statistical power for analyses. Microhistological studies, in contrast, often collect small numbers of samples opportunistically using convenience sampling that suffers spatial bias. Preliminary power analyses revealed that collection of >40 composite samples each summer would be necessary to simply test for changes in the proportions of a single diet item, lichen, in the summer diet of caribou (L. Ehlers, unpublished data). Regardless, this bias in microhistological sampling and low taxonomic resolution are likely responsible for the lower correlation within forage types.

Despite the methodological challenges, the strong tradeoff we observed with videos between shrubs and lichen has important implications for caribou nutritional ecology. Caribou clearly eat shrubs in summer to accumulate fat, because of their relatively high digestibility properties and nitrogen content (Boertje, 1984; Murie, 1935; Skoog, 1956; White et al., 2013). The diet estimates we obtained from video collars support our predictions and match nearly a century of a broad array of different types of studies from Alaska and Canada (Boertje, 1990; Murie, 1935; Russell et al., 1993; Skoog, 1956; Thompson & McCourt, 1981) that documented tradeoffs between shrubs and lichens between seasons and, in our study, within summer. Forbs accounted for small portions of the diet but increased gradually as the growing season advanced. Graminoids were also generally rare (<10%) in caribou diet in early and late summer (Boertje, 1984; Russell et al., 1993; Skoog, 1956). The tradeoff observed from lichen to shrubs occurred when shrubs green up in early summer (June; Figure 5). However, the decline in shrub consumption we observed in July may arise because of insect‐induced shifts in resource selection where caribou select higher elevations, forcing animals to suboptimal habitats where shrub biomass is reduced (Russell et al., 1993; Appendix B Figures B1 and B3). In the future, we can assess how spatial covariates affect diet estimated from video collars; something we have never been able to do with fecal samples. Combined with the evident bias against shrubs in microhistological samples, which are critical for summer protein replenishment (White et al., 2013), we conclude that video collars provide researchers a powerful tool to study changes in caribou diet over time and at fine spatial scales.

### 4.4 Significance and conclusions

High abundance and declining indices of nutritional condition (Boertje et al., 2012) have led to questions about deteriorating summer range conditions, making understanding foraging behavior and diet of the Fortymile Caribou Herd of central importance to management. If the Fortymile Caribou Herd is near ecological carrying capacity, caribou across the population may be forced into lower‐quality habitats during summer. The rise in the proportion of shrubs consumed in the diet we observed, especially in video data, might alleviate concerns about nutritional limitation arising from low‐quality diets (composed of poor‐quality lichen) during the critical summer nutritional window. Willow (*Salix* spp.) may be susceptible to overuse during phases of high caribou abundance, although shrubs can recover quickly from periods of intense grazing. However, both diet methods showed a high diet content of lichen during summer. Macander et al. (2020) showed lichen‐rich habitats were selected by animals in the Fortymile Caribou Herd in both winter and summer. Lichen has a much longer recovery time following destruction, suggesting that if lichen is important for nutritional condition (e.g., Messier et al., 1988), recovery may be delayed when caribou are at higher abundances or if wildfires reduce lichen availability throughout the summer range (Macander et al., 2020). Future studies can further test for spatial tradeoffs between lichen‐rich (e.g., Macander et al., 2017) and shrub‐rich landcover types in summer to understand if density‐dependent habitat selection is driving this tradeoff and to test for potential consequences of foraging in high‐shrub versus high‐lichen habitats for nutritional condition at the individual and population levels. Understanding caribou diet and foraging ecology is needed to plan for their long‐term conservation across the circumpolar north, given the accelerated effects of climate change in these regions and the uncertain future of many caribou herds.

## CONFLICT OF INTEREST

The authors have no conflicts of interests to declare.

## AUTHOR CONTRIBUTIONS

**Libby Ehlers:** Conceptualization (equal); data curation (lead); formal analysis (lead); investigation (equal); methodology (lead); project administration (equal); supervision (equal); visualization (lead); writing–original draft (lead); writing–review and editing (lead). **Gabrielle Coulombe:** Data curation (equal); methodology (supporting); project administration (supporting); writing–original draft (supporting); writing–review and editing (supporting). **Jim Herriges:** Conceptualization (equal); data curation (equal); funding acquisition (lead); investigation (supporting); methodology (supporting); project administration (supporting); resources (equal); writing–review and editing (equal). **Torsten Bentzen:** Conceptualization (equal); data curation (equal); funding acquisition (supporting); investigation (supporting); methodology (supporting); resources (equal); writing–review and editing (equal). **Michael Suitor:** Conceptualization (equal); data curation (equal); funding acquisition (supporting); investigation (supporting); methodology (supporting); resources (equal); writing–review and editing (equal). **Kyle Joly:** Formal analysis (supporting); funding acquisition (supporting); methodology (supporting); resources (supporting); writing–review and editing (equal). **Mark Hebblewhite:** Conceptualization (equal); data curation (supporting); formal analysis (supporting); funding acquisition (supporting); project administration (supporting); resources (supporting); supervision (supporting); writing–review and editing (equal).

## Figures and Tables

**FIGURE B6:**
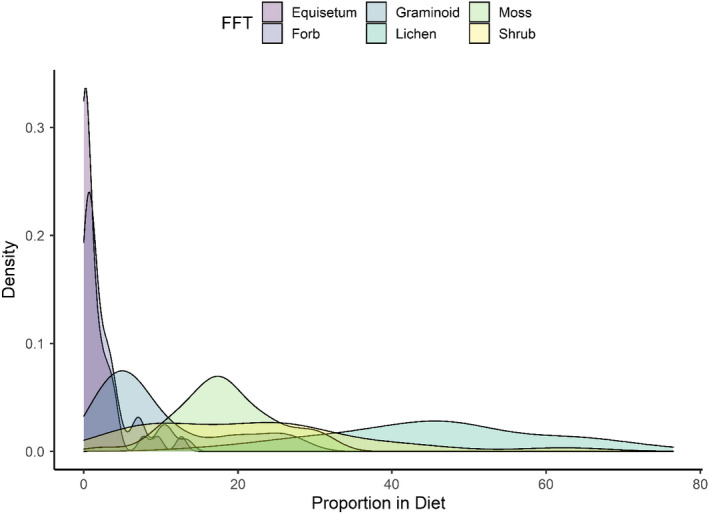
Variation of forage functional types (FFT) in the summer diet of caribou in the Fortymile Caribou Herd based on microhistological analysis

## Data Availability

Data have been deposited in Dryad. https://doi.org/10.5061/dryad.h18931zmz

## APPENDIX A

### FORTYMILE CARIBOU HERD — VIDEO PROCESSING PROTOCOL

Phase 1: Initial Screening

**Table:**

Principal Investigator: Dr. Mark Hebblewhite Project Lead: Libby Ehlers, PhD Candidate Ungulate Ecology Lab Wildlife Biology Program W.A. Franke College of Forestry & Conservation University of Montana	Project Manager: Gabrielle Coulombe Research Associate Stone Hall 108, University of Montana gabrielle.coulombe@umontana.edu 406‐304‐7046

Project Summary: PhD candidate Libby Ehlers is collaborating with the Bureau of Land Management, Alaska Department of Fish & Game, Environment Yukon and the National Park Service to study the Fortymile Caribou Herd in Alaska and Yukon. Forage data were collected via field sampling, remote sensing and animal‐borne GPS collars with built‐in video cameras. This project is part of a larger study on environmental change in the boreal and arctic regions, where warming is occurring at a faster rate than the global average (above.nasa.gov). Video and geospatial data collected from caribou collars will provide information on caribou diet, resource selection, reproductive success and activity budgets.

Video collars were placed on female caribou and programmed to record a 9‐s video clip every 20 min during daylight hours, from May to September. A total of 92,000 video clips were obtained from 15 animals in 2018, with similar numbers in 2019. Video clips are processed in two phases, conducted online:

- Phase 1 (this document) consists of the initial screening of a large subsample of clips. The observer's task is to view each clip and complete a short online form. Clips identified as showing foraging activity are then used in Phase 2 of data collection.
- Phase 2 focuses on caribou diet and requires observers able to identify the Alaska/Yukon flora to the genus taxonomic level. The data collected will then be combined with GPS locations from the video collars and results from field‐based sampling of forage quality and biomass.

#### Observer procedure

1. Sign up (contact the project manager to express your interest)
2. Learn this protocol and evaluate your proficiency (2–3 h in total)
3. Questions/feedback as needed
4. Collect and enter data (~2 min per clip): view video clips and submit forms

#### Requirements

- Computer; speakers/headphones can help detect foraging activity.
- Good internet connection and mainstream web browser.
- **Split screen**: for consistency and efficiency, view the clips on one side of the screen (in one browser window) and the data entry form on the other side (in a separate browser window). Regardless of the device used, **the “video window” should be equivalent to at least half the size of a typical laptop screen**. The “form window” can be made narrower without losing functionality. Please contact the project manager for any help.

#### Viewing video clips

Each qualifying observer is assigned a folder containing a unique set of 90 video clips (six random clips from each of the 15 study animals). Clips are viewed online via a custom link to UM Box (University of Montana's cloud‐based storage). **You may need to view each clip more than once** to focus on the different types of data to be collected. To navigate to the next clip, hover the cursor over the image and click on the arrow. **Video file names contain the animal ID, date, and time: “ID#_YYYYMMDD_HHMMSS”**.

If you have completed your folder and are still available, please contact the project manager and a new folder will be assigned to you. If you are unable to complete your folder, please notify the project manager and the remaining clips will be reassigned.

#### Entering data

Data are entered in Google Forms online. A link to the live form will be provided along with your assigned folder. In the meantime, please follow the link below to preview the form while learning the protocol.

- Form Preview: tinyurl.com/y3y9avap

Use the “NEXT” and “BACK” buttons at the bottom to navigate across the three sections of the form (please avoid using your browser buttons). Upon submitting a form, you may choose to edit your response, fill another form or close the window until your next data entry session. **Please keep track of your progress in order to prevent duplicates or missed entries**. If you lose track of your progress, contact the project manager and you will be pointed in the right direction.

The data collected pertain to the individual caribou wearing the video collar. **Please refer to the video examples and field descriptions below**. If uncertainty remains, enter your best response and then flag the form for review in the last section of the form. You may additionally contact the project manager for a quicker response. If you realize along the way that you have been misinterpreting a question or have not entered the best possible response in previous submissions, let us know and steps will be taken to edit those responses. Please keep in mind that some video clips are ambiguous and **the observer's best assessment is usually sufficient!** However, for reoccurring uncertainties regarding foraging activity or calf identification, please contact us for further guidance.

- Video Examples: tinyurl.com/yc9r67zz

More video examples will be added as we go, so please refer to this folder often through the data collection process. Video file names in the examples indicate the correct assessment for each type of data collected.

**Table:**

FORM — Section 1 of 3

#### Observer Name

For quick navigation through the list: click “Choose”, then scroll down or type the first letter of your first name (keep pressing that same letter to navigate to your name) and press enter.

#### File name

This is **the most important entry** of the form.

1. Locate the file name (top‐left of the video window), select it by double‐clicking (no need to include the file extension, but it can also be included), then press ctrl‐C (Mac: command‐C).
2. Paste into the form: ctrl‐V (command‐V).
3. Please ensure that the file name has copied correctly.

#### Video quality

This is a quick, somewhat arbitrary assessment. See video examples linked on page 2. Camera lens obstruction may consist of long fur, condensation, water drops, dirt etc.

- EXCELLENT — excellent image
- FAIR to GOOD — **most clips fall in this category**; allows easy observation, partial to no camera lens obstruction
- POOR — some data can be collected but the image is problematic (e.g., significant lens obstruction, low light, problematic camera angle and blurry image)
- EXTREMELY OBSTRUCTED — the image is obstructed the entire time (often by the chin or fur while eating) and **a botanist would not be able to identify any of the vegetation present**.

**Table:**

FORM — Section 2 of 3

#### Foraging Status

During summer days, caribou spend almost half of their time eating and a quarter of their time ruminating. Please view the video examples linked on page 2.

- RUMINATING — Caribou are ruminants (like cattle) and spend a lot of time chewing their cud (food that is regurgitated from their first stomach compartment to be chewed a second time). **If the caribou is chewing while bedded or resting, it is almost certainly ruminating**. They can also ruminate while walking if they get disturbed. If you see “swollen” cheeks or the bolus going up the esophagus, the caribou is definitely ruminating. If the cheeks are not bulging, the caribou may nonetheless be ruminating, **please view the video examples!**
- CHEWING — Chewing food but did not take a bite during the video recording (only took a bite before the start of the recording; e.g., chewing while searching for food).
- EATING — “Took a bite” of a food item. **Select “eating” even if you cannot identify the food item consumed** (when the caribou eats, fur from its neck can obstruct the camera; having the sound on can help identify eating activity).
- DRINKING
- LICKING — Licked the soil/rock for minerals but did not take a bite of a food item.
- None of the above

#### State of Locomotion

This field may be ambiguous for some clips, and **your best assessment is sufficient (do not flag for review or comment)**. Please select the first applicable option in the list.

- Wading/Swimming
- Running
- Walking
- Stationary Awake: standing or lying, but awake
- Napping: head on the ground, minimal movement (breathing, twitching), may see curled up legs/hooves or sideways camera angle

#### Is a calf visible?

If age determination is not obvious, please flag for review in the last section of the form.

- Yes — her own: calving evidence (placenta/sac, wet neonate) or maternal behavior (suckling, licking/grooming, being near a very young calf or very close to a young calf)
- Yes — possibly her own: no maternal behavior detected, but the calf is not with another cow
- Yes — calf is with another cow
- No

*Calf* *identification tips:*

- *Look for a smaller body*, *shorter ears and shorter face with a narrower snout*.
- ***Look at the timestamp (YYYYMMDD) in the video file name***. *Calves were born around May 19–28*, *so identification is easier in May–June and becomes progressively more difficult*. ***The example videos can be sorted by date and include non*‐*calves as a comparison***.
- *Caribou color is highly variable and not reliable for age determination*.
- *An antlerless caribou is not necessarily a calf*.
- *Small antlers (spikes) may be visible on calves by late summer*.

#### Other caribou visible (excluding own calf)?

- Yes — herd (about 10 or more caribou)
- Yes — one to a few individuals
- No

#### Does the cow have antlers?

It is sometimes possible to confirm the presence or absence of antlers when the caribou's shadow is visible or to confirm the presence of antlers through a direct glimpse of the top of the caribou's head. There is no need to spend time on assessing this outside the period of May to early June (see date stamp in the file name). **From mid‐June to September, you can simply select “Not relevant”**. Antler presence in May to early June provides an additional clue that a caribou was pregnant, as pregnant cows drop their antlers later than non‐pregnant cows (retaining antlers helps defend food patches later into the season). By fall, all caribou have grown new antlers.

- Yes
- No
- Can't see/Not sure/Not relevant (**most clips fall in this category**)

#### Potential insect harassment behavior (select all that apply)

- Shook its head
- Kept its nose still AND on the ground (to prevent parasitic flies from laying eggs in the nostrils)
- Scratched (may use mouth or hoof)
- Sought snow patch (lying/standing on a snow patch, as opposed to just walking or foraging through snow)
- Huddled
- None of the above

#### What part of the habitat is visible?

- Ground and immediate surroundings (a good glimpse of the habitat is sufficient, as long as the predominant vegetation type around the caribou can be identified)
- Only ground
- None

#### What is the PREDOMINANT vegetation?

Quick assessment of the main vegetation type present **near the caribou**. Any category (including poor visibility) may be selected on their own or in concurrence with another. Select only the **predominant** categories (preferably 1 or 2, but can be up to 3).

- Poor Visibility: this video clip offers poor visibility of the predominant vegetation
- Alpine Tundra: high elevation/latitude prevents tree growth; vegetation grows close to the ground and consists mainly of grasses, sedges and forbs, and may include lichen, dwarf woody or semi‐woody shrubs, or mosses
- Lichen/Moss/Herbaceous (“herbaceous” includes grasses and forbs/flowering plants)
- Shrubby: small‐ to medium‐sized woody plant, excluding coniferous saplings
- Forested – Deciduous
- Forested – Coniferous
- Unvegetated Areas: rocks, water

*Vegetation assessment tips*: *Alpine tundra can be thought of as “open habitat” (where trees cannot grow to maturity*, *because of the high elevation*, *low moisture*, *poor soil*, *cold and often windy conditions)*. *Selecting “lichen*/*moss*/*herbaceous” would also be correct*, *but when the surrounding open and dry habitat is visible*, *“alpine tundra” is more precise*. *You may also encounter “lichen*/*moss*/*herbaceous” vegetation outside alpine tundra*, *for example*, *on a forest floor or unknown location (sometimes the clip does not show the wider habitat)*, *so it is also possible to select “lichen*/*moss*/*herbaceous” on its own or in combination with forest*, *poor visibility etc*.

#### Habitat features visible (select all that apply)

- Snow cover 1%–50% (in the vicinity, ignore mountain tops and faraway snow)
- Snow cover 50%–100% (in the vicinity, ignore mountain tops and faraway snow)
- Water (e.g., river and puddle)
- Burn area visible (at any successional stage; e.g., burn scars and sooty snags/logs)
- Human signs: any sign of human presence (e.g., human activity, roads, buildings and other structures)
- None of the above

**Table:**

FORM — Section 3 of 3

#### Other species detected?

Enter the type of animal detected (e.g., mammal, canine, bird and bird of prey) or finer taxonomic level if known (e.g., wolf and golden eagle).

#### FLAG for review? “There was uncertainty in my response(s) regarding…”

Some footage may be difficult to interpret, and a second opinion will help determine the best response(s). Please note that **the observer's best assessment is usually sufficient** without need for review. However, for reoccurring uncertainties, particularly regarding eating or calf identification, please communicate with us for further guidance.

- Ruminating/Chewing/Eating
- Calf identification
- Maternal behavior/Calving evidence
- Other: **(additional comments can be added here)**

#### FLAG as favorite? “This clip is an outstanding example of…”

Please select all reasons that apply. More details or categories can be added under “Other”.

- Potential predation attempt (rare video capture, please flag!)
- Interesting/rare behavior or interaction
- Interesting vegetation/habitat feature
- Visually appealing video clip (e.g., scenery, herd, calf and habitat)
- Sounds (e.g., caribou call and other species). Please do not flag ruminating sounds and sounds of vegetation rubbing against the collar.
- Other: (additional comments can be added here)

#### Note (Please use very sparingly)

This field may be used to relay pertinent information not otherwise included in the form. Please be concise, use key words and avoid repeating information already entered. **Almost always leave this field blank!**

*Tips for writing notes*: *It is important to only write a comment in this section if there is something particularly extraordinary or peculiar and leave it blank otherwise*. *The bulk of the data needed is already included in the form*.

### TRAINING

The training procedure is conducted online and through communication with peers or project contacts. We aim to ensure consistency and efficiency among observers, generate high quality data and provide a platform for questions and feedback, which may help improve the data collection process.

Once you have read the field descriptions above and viewed the video examples, please study each pre‐filled form below and read the “practice notes” at the end of each form. Questions and feedback are welcome at any time.

- Videos for prefilled forms: tinyurl.com/y2wsgj6q

**Table:**

Video File Name	Pre‐filled Form
01_1154_20180908_194901	tinyurl.com/y2bhzg4l
02_1154_20180909_172900	tinyurl.com/y6b2c2ny
03_1170_20180520_231021	tinyurl.com/y492h5nt
04_1155_20180906_022838	tinyurl.com/yycpdje5
05_1159_20180908_210900	tinyurl.com/y3uz8o8u
06_1173_20180831_034902	tinyurl.com/y2g38hvc
07_1136_20180511_031006	tinyurl.com/yy4ab7h7
08_1170_20180511_221052	tinyurl.com/y678la7u
09_1155_20180610_192922	tinyurl.com/yyzufa87
10_1136_20180521_174958	tinyurl.com/y24j2k82

### EVALUATION

Please submit an evaluation form for each of the 10 evaluation videos in the folder linked below.

- Evaluation Videos: tinyurl.com/y54dck83
- Evaluation Form: forms.gle/1d1MKgz4bpDC4pXg6

Once the task has been completed, please notify the project manager to discuss your results and receive your assigned folder. Thank you for your interest in being part of this project! (Tables B3, B5, B6 and Figure B6)

## APPENDIX B

### Foraging ecology and diet analysis

**TABLE B1:** Possible combinations of eating and insect avoidance behaviors observed and classified in videos

Eating	Insects	# of observations	% of total observations
0	0	9,251	51.0
0	1	1,002	5.5
1	0	7,778	42.9
1	1	103	0.6

Note

We classified a total of 18,134 videos over two summers (2018 and 2019) into different behavioral activity states. The variables representing “Eating” and “Insects” represent a binary outcome where an observation received a “1” if a caribou was observed consuming forage. Similarly, if a caribou was observed displaying insect avoidance behavior(s), “Insects = 1”.

**TABLE B2:** Candidate models to test for relationship between the frequency of eating and insect avoidance behaviors

Model #	Name	Description of model components
1	Null (no relationships)	
2	Insects	Fixed effects
3	Month	
4	Year	
5	CamID_Yr	
6	Month + Year	
7	Month * Year	
8	Insects + Year	
9	Insects * Year	
10	Insects + Month	
11	Insects * Month	
12	Insects + CamID_Yr	Covariate model w/ fixed effect of individual
13	Insects + Year + Insects * Year	
14	Insects + Month + Insects * Month	
15	Insects + Year + Month	
16	Insects + Year + Month + Insects * Year + Insects * Month	
17	Insects + (1 | CamID_Yr)	No random effects; random group intercept for individual female
18	Insects + (0 + Insects | CamID_Yr)	Random covariate
19	Insects + (Insects | CamID_Yr)	Random intercept and covariate
20	Insects + Month + Year + Insects * Year + Insects * Month + (1 | CamID_Yr)	Mixed effects model w/ random intercept
21	Insects + MonthF + (1 | CamID_Yr)	Mixed effects model w/ random intercept
22	Insects + MonthF + Insects * MonthF + (1 | CamID_Yr)	Mixed effects model w/ random intercept
23	Insects + MonthF + YearB + Insects * YearB + (1 | CamID_Yr)	Mixed effects model w/ random intercept

**TABLE B3:** Taxonomic resolution of videos classified to assess the summer diet for females (*n* = 30) of the Fortymile Caribou Herd

*Taxonomic level*	Number of videos	Proportion of videos
Family	188	2.50%
Genus	2,386	31.69%
FFT	1,151	15.29%
FFT unidentifiable	1,379	18.32%
Species	2,425	32.21%
**Grand total**	**7,529**	**100.00%**

Note

Five botanists reviewed videos (*n* = 5,549) of caribou eating to identify the forages consumed (*n* = 7,529). We categorized classified forage videos into the following taxonomic levels: family, genus, forage functional type (FFT), forage functional type unidentifiable (FFT unidentifiable) and species.

**TABLE B4:** Apparent dry matter digestibility (DMD% in g/g) of summer diet for caribou in the Fortymile Caribou Herd (*Rangifer tarandus granti*)

Forage types	Apparent dry matter digestibility (DMD; g/g)	Correction factor	Sample size	Notes
Forb	0.77	0.23	8	No *Equisetum* spp. included mostly lupine, fireweed and anenome
Graminoid (incl *Carex* spp.)	0.73	0.27	16	
Lichen	0.75	0.25	12	
Shrubs	0.58	0.42	82	Deciduous shrubs

Note

We measured apparent dry‐matter digestibility (DMD%; Van Soest, 1982) for plants at the levels of family, genus, forage functional type (FFT), forage functional type unidentifiable (FFT unidentifiable) and species, to correct fecal diet samples for digestibility. Correcting for digestibility facilitated comparison of video‐ to fecal‐derived diet estimates.

**TABLE B5:** Complete plant list as identified by GPS video‐camera collars

FFT	Family	Genus	Final ID	Taxonomic level	Common name	# clips 2018	# clips 2019	# clips total	% clips 2018	% clips 2019	% clips total
Equisetum	Equisetaceae	Equisetum	**Equisetum**	**Genus**	horsetail	52	7	59	1.17	0.63	1.06
Equisetum	Equisetaceae	Equisetum	**Equisetum scirpoides**	**Species**	dwarf scouring rush, dwarf horsetail	1		1	0.02	0	0.02
Forb	Apiaceae	Bupleurum	**Bupleurum**	**Genus**		1		1	0.02	0	0.02
Forb	Apiaceae	Heracleum	**Heracleum lanatum**	**Species**	cow parsnip	1		1	0.02	0	0.02
Forb	Asteraceae	Arnica	**Arnica**	**Genus**		4	1	5	0.09	0.09	0.09
Forb	Asteraceae	Artemisia	**Artemisia**	**Genus**	mugwort, wormwood, sagebrush	3	3	6	0.07	0.27	0.11
Forb	Asteraceae	Artemisia	**Artemisia arctica/norvegica**	**Species**	sagewort, mugwort, wormwood	22	6	28	0.49	0.54	0.5
Forb	Asteraceae	Petasites	**Petasites**	**Genus**	coltsfoots, butterburs	4		4	0.09	0	0.07
Forb	Asteraceae	Petasites	**Petasites frigidus**	**Species**	arctic sweet coltsfoot, arctic butterbur	29	1	30	0.65	0.09	0.54
Forb	Asteraceae	Saussurea	**Saussurea angustifolia**	**Species**	narrowleaf saw‐wort	8	3	11	0.18	0.27	0.2
Forb	Asteraceae	Solidago	**Solidago multiradiata**	**Species**	Rocky Mountain goldenrod, northern goldenrod, alpine goldenrod	1		1	0.02	0	0.02
Forb	Asteraceae		**Asteraceae**	**Family**	Compositae; aster, daisy, composite, or sunflower family	3	2	5	0.07	0.18	0.09
Forb	Boraginaceae	Mertensia	**Mertensiapaniculata**	**Species**	tall lungwort, tall bluebells, northern bluebells	3	1	4	0.07	0.09	0.07
Forb	Brassicaceae	Cardamine	**Cardamine purpurea**	**Species**	purple bittercress		1	1	0	0.09	0.02
Forb	Caprifoliaceae/Valerianaceae	Valeriana	**Valeriana capitata**	**Species**		2	1	3	0.04	0.09	0.05
Forb	Caryophyllaceae		**Caryophyllaceae**	**Family**		1		1	0.02	0	0.02
Forb	Ericaceae	Pyrola	**Pyrola**	**Genus**	wintergreen	2		2	0.04	0	0.04
Forb	Fabaceae	Astragalus	**Astragalus**	**Genus**	milkvetch, locoweed, goat's‐thorn	2		2	0.04	0	0.04
Forb	Fabaceae	Astragalus/Hedysarum	**Astragalus/Hedysarum**	**Genus**		4		4	0.09	0	0.07
Forb	Fabaceae	Astragalus/Oxytropis	**Astragalus/Oxytropis**	**Genus**		3	2	5	0.07	0.18	0.09
Forb	Fabaceae	Hedysarum	**Hedysarum**	**Genus**	sweetvetch	1	1	2	0.02	0.09	0.04
Forb	Fabaceae	Lupinus	**Lupinus**	**Genus**	lupine, lupin	6	4	10	0.13	0.36	0.18
Forb	Fabaceae		**Fabaceae**	**Family**	Leguminosae, legume, pea, bean family	11	3	14	0.25	0.27	0.25
Forb	Liliaceae	Lloydia	**Lloydia serotina**	**Species**	Gagea serotina, Snowdonalplily, mountain spiderwort	1		1	0.02	0	0.02
Forb	Liliaceae		**Liliaceae**	**Family**	lily family	1		1	0.02	0	0.02
Forb	Onagraceae	Chamaenerion	**Chamaenerion angustifolium**	**Species**	fireweed, great willowherb, Chamerion/Epilobium angustifolium	17	9	26	0.38	0.82	0.47
Forb	Onagraceae	Chamaenerion	**Chamaenerionlatifolium**	**Species**	dwarf fireweed, river beauty willowherb	1	4	5	0.02	0.36	0.09
Forb	Onagraceae		**Onagraceae**	**Family**	willowherb, evening primrose family	1		1	0.02	0	0.02
Forb	Orobanchaceae	Pedicularis	**Pedicularis**	**Genus**	lousewort	8	5	13	0.18	0.45	0.23
Forb	Orobanchaceae	Pedicularis	**Pedicularisoederi**	**Species**	Oeder's lousewort	1		1	0.02	0	0.02
Forb	Polygonaceae	Bistorta	**Bistorta**	**Genus**		17	2	19	0.38	0.18	0.34
Forb	Polygonaceae	Bistorta	**Bistorta plumosa**	**Species**	meadow bistort, pink plumes	1	1	2	0.02	0.09	0.04
Forb	Polygonaceae	Oxyria	**Oxyriadigyna**	**Species**	mountain sorrel, wood sorrel, Alpine sorrel	2		2	0.04	0	0.04
Forb	Polygonaceae	Polygonum	**Polygonum**	**Genus**	knotweed, knotgrass	6		6	0.13	0	0.11
Forb	Polygonaceae	Rumex	**Rumex**	**Genus**	docks, sorrels	2		2	0.04	0	0.04
Forb	Polygonaceae	Rumex	**Rumex arcticus**	**Species**	arctic dock, sourdock	3		3	0.07	0	0.05
Forb	Polygonaceae		**Polygonaceae**	**Family**	buckwheat, smartweed, knotweed	5		5	0.11	0	0.09
Forb	Primulaceae	Dodecatheon	**Dodecatheon**	**Genus**	shooting star, American cowslip, mosquito bills, mad violets, sailor caps	2		2	0.04	0	0.04
Forb	Primulaceae	Dodecatheon	**Dodecatheon frigidum**	**Species**	western arctic shootingstar		1	1	0	0.09	0.02
Forb	Ranunculaceae	Aconitum	**Aconitum delphinifolium**	**Species**	northern monkshood	13	2	15	0.29	0.18	0.27
Forb	Ranunculaceae	Anemone	**Anemone**	**Genus**		12	7	19	0.27	0.63	0.34
Forb	Ranunculaceae	Anemone	**Anemone narcissiflora**	**Species**	narcissus anemone	8	6	14	0.18	0.54	0.25
Forb	Ranunculaceae	Anemone	**Anemone parviflora**	**Species**	northern anemone, small‐flowered anemone	10		10	0.22	0	0.18
Forb	Ranunculaceae	Ranunculus	**Ranunculus**	**Genus**	buttercups, spearworts, water crowfoots	4	1	5	0.09	0.09	0.09
Forb	Ranunculaceae		**Ranunculaceae**	**Family**	buttercup, crowfoot family; Ranunculus, Delphinium, Thalictrum, Clematis, Aconitum, etc.	23	5	28	0.52	0.45	0.5
Forb	Rosaceae	Dasiphora/Potentilla	**Dasiphora/Potentilla**	**Genus**	cinquefoil	1		1	0.02	0	0.02
Forb	Rosaceae	Rubus	**Rubus arcticus/chamaemorus**	**Species**		1		1	0.02	0	0.02
Forb	Rosaceae	Rubus	**Rubus chamaemorus**	**Species**	aqpik, low‐bush salmonberry (not to be confused with true salmonberry, Rubus spectabilis, cloudberry)	2	1	3	0.04	0.09	0.05
Forb	Rubiaceae	Galium	**Galiumboreale**	**Species**	northern bedstraw	1		1	0.02	0	0.02
Forb	Saxifragaceae	Boykinia	**Boykinia**	**Genus**	brookfoams	1	1	2	0.02	0.09	0.04
Forb	Saxifragaceae	Boykinia	**Boykiniarichardsonii**	**Species**	bear flower	13	9	22	0.29	0.82	0.4
Forb	Saxifragaceae	Saxifraga	**Saxifraga**	**Genus**	saxifrages, rockfoils	3	1	4	0.07	0.09	0.07
Forb	Saxifragaceae	Saxifraga	**Saxifraga nelsoniana**	**Species**	heartleaf saxifrage	1	1	2	0.02	0.09	0.04
Forb	Saxifragaceae		**Saxifragaceae**	**Family**		1		1	0.02	0	0.02
Forb	Unknown forb		**Unknown forb**	**FFT**		90	22	112	2.02	1.99	2.02
Graminoid	Cyperaceae	Carex	**Carex**	**Genus**	true sedges	117	29	146	2.63	2.63	2.63
Graminoid	Cyperaceae	Carex	**Carexbigelowii**	**Species**	Bigelow's sedge, Gwanmo sedge, stiff sedge	12	7	19	0.27	0.63	0.34
Graminoid	Cyperaceae	Carex	**Carexmicrochaeta**	**Species**	smallawned sedge		1	1	0	0.09	0.02
Graminoid	Cyperaceae	Eriophorum	**Eriophorum**	**Genus**	cottongrass, cottonsedge	19	8	27	0.43	0.72	0.49
Graminoid	Cyperaceae	Eriophorum	**Eriophorum angustifolium**	**Species**	common cottongrass, common cottonsedge	4		4	0.09	0	0.07
Graminoid	Cyperaceae	Eriophorum	**Eriophorumvaginatum**	**Species**	hare's‐tail/tussock cottongrass, sheathed cottonsedge	47	13	60	1.06	1.18	1.08
Graminoid	Cyperaceae		**Cyperaceae**	**Family**	Sedges	17	6	23	0.38	0.54	0.41
Graminoid	Juncaceae		**Juncaceae**	**Family**	Rushes	1		1	0.02	0	0.02
Graminoid	Poaceae	Arctagrostis	**Arctagrostis latifolia**	**Species**	broad‐leaf arctic‐bent, polar grass, wideleafpolargrass	2		2	0.04	0	0.04
Graminoid	Poaceae	Calamagrostis	**Calamagrostis**	**Genus**	reed grass, smallweed	2	1	3	0.04	0.09	0.05
Graminoid	Poaceae	Calamagrostis	**Calamagrostis canadensis**	**Species**	bluejoint, reed grass, meadow/marsh pinegrass	1	1	2	0.02	0.09	0.04
Graminoid	Poaceae	Festuca	**Festuca altaica**	**Species**	altai fescue, Festuca scabrella (rough fescue)	11	13	24	0.25	1.18	0.43
Graminoid	Poaceae	Hierochloe	**Hierochloealpina**	**Species**	alpine sweetgrass, Anthoxanthummonticola	1	2	3	0.02	0.18	0.05
Graminoid	Poaceae		**Poaceae**	**Family**	grasses	49	15	64	1.1	1.36	1.15
Graminoid	Unknown graminoid		**Unknown graminoid**	**FFT**	grasses/sedges/rushes	98	29	127	2.2	2.63	2.29
Lichen	Cladoniaceae	Cladina	**Cladina**	**Genus**	reindeer lichens, forage lichens, mat‐forming lichens	259	106	365	5.83	9.6	6.58
Lichen	Cladoniaceae	Cladina/Cladonia	**Cladina/Cladonia**	**Genus**		382	135	517	8.59	12.23	9.32
Lichen	Cladoniaceae	Cladina/Cladonia	**Cladina/Cladoniarangiferina/stygia**	**Species**	reindeer lichen, reindeer moss, caribou moss; Lichen rangiferinus	169	41	210	3.8	3.71	3.78
Lichen	Cladoniaceae	Cladonia	**Cladonia**	**Genus**	cup lichen	10	10	20	0.22	0.91	0.36
Lichen	Cladoniaceae	Cladonia	**Cladonia mitis**	**Species**	C. arbuscula subsp. mitis, green reindeer lichen	16	2	18	0.36	0.18	0.32
Lichen	Cladoniaceae	Cladonia	**Cladonia stellaris**	**Species**		3	2	5	0.07	0.18	0.09
Lichen	Cladoniaceae		**Cladoniaceae**	**Family**	reindeer moss, cup lichens	7	4	11	0.16	0.36	0.2
Lichen	Icmadophilaceae	Thamnolia	**Thamnolia**	**Genus**	whiteworm lichens	13	5	18	0.29	0.45	0.32
Lichen	Icmadophilaceae	Thamnolia	**Thamnolia vermicularis**	**Species**		12	11	23	0.27	1	0.41
Lichen	Icmadophilaceae		**Icmadophilaceae**	**Family**			1	1	0	0.09	0.02
Lichen	Nephromataceae	Nephroma	**Nephroma**	**Genus**	kidney lichens	1		1	0.02	0	0.02
Lichen	Parmeliaceae	Cetraria	**Cetraria**	**Genus**	syn. Coelocaulon	3		3	0.07	0	0.05
Lichen	Parmeliaceae	Cetraria	**Cetraria**	**Genus**		2		2	0.04	0	0.04
Lichen	Parmeliaceae	Cetraria/Dactylina	**Cetraria/Dactylina**	**Genus**		1		1	0.02	0	0.02
Lichen	Parmeliaceae	Dactylina	**Dactylina**	**Genus**		4		4	0.09	0	0.07
Lichen	Parmeliaceae	Evernia	**Evernia**	**Genus**		1		1	0.02	0	0.02
Lichen	Parmeliaceae	Flavocetraria	**Flavocetraria**	**Genus**		78	14	92	1.75	1.27	1.66
Lichen	Parmeliaceae	Flavocetraria	**Flavocetraria nivalis**	**Species**		1		1	0.02	0	0.02
Lichen	Parmeliaceae	Flavocetraria/Cetraria	**Flavocetraria/Cetrariacucullata**	**Species**		141	53	194	3.17	4.8	3.5
Lichen	Parmeliaceae	Masonhalea	**Masonhalearichardsonii**	**Species**		2	1	3	0.04	0.09	0.05
Lichen	Parmeliaceae		**Parmeliaceae**	**Family**		2		2	0.04	0	0.04
Lichen	Sphaerophoraceae	Sphaerophorus	**Sphaerophorus**	**Genus**	ball lichens, coral lichens, tree coral	11	1	12	0.25	0.09	0.22
Lichen	Sphaerophoraceae		**Sphaerophoraceae**	**Family**		1		1	0.02	0	0.02
Lichen	Stereocaulaceae	Stereocaulon	**Stereocaulon**	**Genus**	snow lichens	11	1	12	0.25	0.09	0.22
Lichen	Stereocaulaceae		**Stereocaulaceae**	**Family**		1		1	0.02	0	0.02
Lichen	Unknown lichen		**Unknown lichen**	**FFT**		102	13	115	2.29	1.18	2.07
Lichen	Unknown white/light macrolichen		**Unknown white/light macrolichen**	**FFT**		531	110	641	11.95	9.96	11.55
Moss	Lycopodiaceae	Lycopodium	**Lycopodium**	**Genus**	clubmosses, ground pines, creeping cedars	1		1	0.02	0	0.02
Moss	Polytrichaceae	Polytrichum	**Polytrichum**	**Genus**	haircap moss, hair moss	1		1	0.02	0	0.02
Moss	Sphagnaceae	Sphagnum	**Sphagnum**	**Genus**		1		1	0.02	0	0.02
Moss	Sphagnaceae	Sphagnum	**Sphagnum**	**Genus**	peat moss	1		1	0.02	0	0.02
Moss	Unknown moss		**Unknown moss**	**FFT**		1	1	2	0.02	0.09	0.04
Mushroom	Boletaceae	Leccinum	**Leccinum**	**Genus**		2	2	4	0.04	0.18	0.07
Mushroom	Boletaceae		**Boletaceae**	**Family**	boletes	1		1	0.02	0	0.02
Mushroom	Russulaceae	Lactarius	**Lactarius**	**Genus**	milk‐caps		1	1	0	0.09	0.02
Mushroom	Unknown mushroom		**Unknown mushroom**	**FFT**		15	25	40	0.34	2.26	0.72
Shrub	Betulaceae	Alnus	**Alnus**	**Genus**	alder	6		6	0.13	0	0.11
Shrub	Betulaceae	Alnus	**Alnus viridis**	**Species**	green alder	1		1	0.02	0	0.02
Shrub	Betulaceae	Betula	**Betula**	**Genus**	birch	26	2	28	0.58	0.18	0.5
Shrub	Betulaceae	Betula	**Betula nana/glandulosa**	**Species**	dwarf birch	589	121	710	13.25	10.96	12.8
Shrub	Betulaceae	Betula	**Betula neoalaskana**	**Species**	B. resinifera, Alaska birch, Alaska paper birch, resin birch	26	8	34	0.58	0.72	0.61
Shrub	Betulaceae	Betula	**Betula occidentalis**	**Species**	water birch, red birch	10	6	16	0.22	0.54	0.29
Shrub	Betulaceae		**Betulaceae**	**Family**	birch family (birch, alders, hazels, hornbeams)	13	3	16	0.29	0.27	0.29
Shrub	Diapensiaceae	Diapensia	**Diapensialapponica/obovata**	**Species**	pincushion plant		2	2	0	0.18	0.04
Shrub	Elaeagnaceae	Shepherdia	**Shepherdia**	**Genus**	buffaloberry, bullberry		1	1	0	0.09	0.02
Shrub	Ericaceae	Andromeda	**Andromeda polifolia**	**Species**	bog‐rosemary	1		1	0.02	0	0.02
Shrub	Ericaceae	Arctostaphylos	**Arctostaphylos**	**Genus**	manzanitas/bearberries		2	2	0	0.18	0.04
Shrub	Ericaceae	Arctostaphylos	**Arctostaphylos rubra/alpina**	**Species**	bearberry, red manzanita, ravenberry, Arctousalpina	27	7	34	0.61	0.63	0.61
Shrub	Ericaceae	Cassiope	**Cassiope**	**Genus**	heath, heather	6	1	7	0.13	0.09	0.13
Shrub	Ericaceae	Empetrum	**Empetrum nigrum**	**Species**	crowberry, blackberry	9	1	10	0.2	0.09	0.18
Shrub	Ericaceae	Kalmia/Loiseleuria	**Kalmia/Loiseleuria procumbens**	**Species**	azalea	1		1	0.02	0	0.02
Shrub	Ericaceae	Rhododendron/Ledum	**Rhododendron groenlandicum/Ledum palustre**	**Species**	bog Labrador tea, formerly Ledum groenlandicum/palustre/latifolium	5		5	0.11	0	0.09
Shrub	Ericaceae	Vaccinium	**Vaccinium**	**Genus**	cranberry, blueberry, bilberry (whortleberry), lingonberry	7	3	10	0.16	0.27	0.18
Shrub	Ericaceae	Vaccinium	**Vaccinium uliginosum**	**Species**	bog bilberry, bog blueberry, northern bilberry, western blueberry	124	52	176	2.79	4.71	3.17
Shrub	Ericaceae	Vaccinium	**Vaccinium uliginosum**	**Species**		1		1	0.02	0	0.02
Shrub	Ericaceae	Vaccinium	**Vaccinium vitis‐idaea**	**Species**	lingonberry, partridgeberry, mountain cranberry, cowberry	12	2	14	0.27	0.18	0.25
Shrub	Ericaceae	Vaccinium	**Vaccinium vitis‐idaea**	**Species**	lingonberry, partridgeberry, mountain cranberry, cowberry	1		1	0.02	0	0.02
Shrub	Ericaceae		**Ericaceae**	**Family**	heath or heather family; cranberry, blueberry, huckleberry, rhododendron (including azaleas), Erica, Cassiope, Daboecia, Calluna	5		5	0.11	0	0.09
Shrub	Rosaceae	Dasiphora/Potentilla	**Dasiphora/Potentilla**	**Genus**	cinquefoil	1		1	0.02	0	0.02
Shrub	Rosaceae	Dasiphora/Potentilla	**Dasiphora/Potentilla fruticosa**	**Species**	shrubby cinquefoil, golden hardhack, bush cinquefoil, shrubby five‐finger, tundra rose, widdy	2		2	0.04	0	0.04
Shrub	Rosaceae	Dryas	**Dryas**	**Genus**		28	16	44	0.63	1.45	0.79
Shrub	Rosaceae	Dryas	**Dryas drummondii**	**Species**	Yellow mountain avens	1		1	0.02	0	0.02
Shrub	Rosaceae	Dryas	**Dryas octopetala**	**Species**	mountain avens, white dryad	28	24	52	0.63	2.17	0.94
Shrub	Rosaceae	Rubus	**Rubus**	**Genus**	raspberries, blackberries, dewberries, etc.		1	1	0	0.09	0.02
Shrub	Rosaceae	Rubus	**Rubus arcticus/chamaemorus**	**Species**		1		1	0.02	0	0.02
Shrub	Rosaceae	Rubus	**Rubus chamaemorus**	**Species**	aqpik, low‐bush salmonberry (not to be confused with true salmonberry, Rubus spectabilis, cloudberry)	1		1	0.02	0	0.02
Shrub	Rosaceae	Spiraea	**Spiraea stevenii**	**Species**	beauverd spirea	1	1	2	0.02	0.09	0.04
Shrub	Salicaceae	Populus	**Populus**	**Genus**	poplar, aspen, cottonwood	2		2	0.04	0	0.04
Shrub	Salicaceae	Populus	**Populus balsamifera**	**Species**	balsam poplar, bam, hackmatack, tacamahac poplar, tacamahaca	7		7	0.16	0	0.13
Shrub	Salicaceae	Populus	**Populus tremuloides**	**Species**	trembling aspen, quaking aspen, white poplar	19	6	25	0.43	0.54	0.45
Shrub	Salicaceae	Salix	**Salix**	**Genus**	willows, osiers, sallows	719	159	878	16.18	14.4	15.82
Shrub	Salicaceae	Salix	**Salix alaxensis**	**Species**	Alaska willow, feltleaf willow	5		5	0.11	0	0.09
Shrub	Salicaceae	Salix	**Salix arctica**	**Species**	arctic willow	5	5	10	0.11	0.45	0.18
Shrub	Salicaceae	Salix	**Salix arctica/phlebophylla/rotundifolia/reticulata**	**Species**	dwarf willows	24	18	42	0.54	1.63	0.76
Shrub	Salicaceae	Salix	**Salix arctica/phlebophylla/rotundifolia/reticulata**	**Species**		2		2	0.04	0	0.04
Shrub	Salicaceae	Salix	**Salix bebbiana**	**Species**	beaked willow, long‐beaked willow, gray willow, Bebb's willow, red willow	5	1	6	0.11	0.09	0.11
Shrub	Salicaceae	Salix	**Salix brachycarpa var. niphoclada**	**Species**	barren‐ground willow, snow willow	1		1	0.02	0	0.02
Shrub	Salicaceae	Salix	**Salix chamissonis**	**Species**	Chamisso's willow	1		1	0.02	0	0.02
Shrub	Salicaceae	Salix	**Salix glauca**	**Species**	gray willow, grayleaf willow, white willow, glaucous willow	10	2	12	0.22	0.18	0.22
Shrub	Salicaceae	Salix	**Salix phlebophylla/rotundifolia**	**Species**	skeleton willow, skeleton‐leaf willow, mountain roundleaf willow, round‐leaved willow		1	1	0	0.09	0.02
Shrub	Salicaceae	Salix	**Salix pulchra**	**Species**	diamondleaf/tealeaf willow, thin red willow; S. planifolia subsp. Pulchra	358	95	453	8.05	8.61	8.16
Shrub	Salicaceae	Salix	**Salix reticulata**	**Species**	net‐leaved willow, snow willow	15	11	26	0.34	1	0.47
Shrub	Salicaceae	Salix	**Salix richardsonii**	**Species**	Richardson's willow, woolly willow	9		9	0.2	0	0.16
Shrub	Salicaceae	Salix	**Salix scouleriana**	**Species**	Scouler's willow; S. brachystachys, S. capreoides, S. flavescens, S. nuttallii, S. stagnalis	1		1	0.02	0	0.02
Shrub	Salicaceae		**Salicaceae**	**Family**	willow family (willows, poplar, aspen, cottonwoods)	4	2	6	0.09	0.18	0.11
Shrub	Unknown dwarf shrub		**Unknown dwarf shrub**	**FFT**		21	18	39	0.47	1.63	0.7
Shrub	Unknown shrub		**Unknown shrub**	**FFT**		46	8	54	1.03	0.72	0.97
Shrub	Unknown tall shrub		**Unknown tall shrub**	**FFT**		19	2	21	0.43	0.18	0.38
Unidentifiable			**Unidentifiable**	**FFT unidentifiable**		5		5	0.11	0	0.09
Unidentifiable ground‐level vegetation			**Ground‐level vegetation**	**FFT unidentifiable**		579	119	698	13.03	10.78	12.58
Unidentifiable ground‐level vegetation, likely lichen			**Likely lichen**	**FFT unidentifiable**		548	128	676	12.33	11.59	12.18

**TABLE B6:** Complete plant list as identified by microhistological analysis of fecal pellet samples

ID#	Full name	Forage functionaltype (FFT)	6 Letter code	Taxon level
1	Agropyron	Grams		
2	Bromus inermis	Grams	BROINE	Spp
3	Calamagrostis canadensis	Grams	CALCAN	Spp
4	Carex spp.	Grams	CAREX	Genus
5	Elymus spp.	Grams	ELYMUS	Genus
6	Eriophorum spp.	Grams	ERIOPH	Genus
7	Festuca altaica	Grams	FESALT	Spp
8	Anthoxanthummonticola (Hierochloealpina)	Grams	ANTMON	Spp
9	Juncus spp.	Grams	JUNCUS	Genus
10	Koeleria macrantha	Grams	KOEMAC	Spp
11	Luzula spp.	Grams	LUZULA	Genus
12	Poa spp.	Grams	POA	Genus
13	Trisetum spicatum	Grams	TRISPI	Spp
14	Unknown Grass	Grams	UKNGRA	PFG
15	Alnus spp.	Shrub	ALNUS	Genus
16	Arctostaphylos rubra/alpina	Shrub	ARCRUB	Spp
17	Artemisia arctica	Shrub	ARTARC	Spp
18	Betula nana/glandulosa	Shrub	BETNANL	Spp
19	Cassiope	Shrub	CASSIO	Genus
20	Diapensialapponica	Shrub	DIALAP	Spp
21	Dryas spp.	Shrub	DRYASL	Genus
22	Empetrum nigrum	Shrub	EMPNIGL	Spp
23	Kalmia polifolia	Shrub	KALPOL	Spp
24	Ledum groenlandicum/palustre	Shrub	LEDGRO	Spp
25	Loiseleuria procumbens	Shrub	LOIPROL	Spp
26	Populus tremuloides	Shrub	POPTREL	Spp
27	Rhododendron spp.	Shrub	RHODOD	Genus
28	Rubus chamaemorus	Shrub	RUBCHA	Spp
29	Rubus spp.	Shrub	RUBUS	Genus
30	Salix spp.	Shrub	SALIXL	Genus
31	Vaccinium vitis‐idaea	Shrub	VACVITL	Spp
32	Unkn shrub	Shrub	UKNSHR	PFG
33	Artemisia spp.	Forb	ARTEMI	Genus
34	Astragalus	Forb	ASTRAG	Genus
35	Chamerion angustifolium	Forb	CHAANG	Spp
36	Equisetum	Forb	EQUISET	Genus
37	Geum	Forb	GEUM	Genus
38	Lupinus	Forb	LUPINU	Genus
39	Mertensia	Forb	MERTEN	Genus
40	Pedicularis	Forb	PEDICUL	Genus
41	Petasites	Forb	PETASI	Genus
42	Polygonum	Forb	POLYGO	Genus
43	Potentilla	Forb	POTENT	Genus
44	Ranunculus	Forb	RANUNC	Genus
45	Sanguisorba officialis	Forb	SANOFF	Spp
46	Saxifraga	Forb	SAXIFRA	Genus
47	Stellaria	Forb	STELLA	Genus
48	Streptopus	Forb	STREPT	Genus
49	Unkn Forb	Forb	UKNFOR	PFG
50	Mushrooms	Mush	MUSHRO	PFG
51	Alectoria/Bryoria/Usnea	Lichen	ALBRYUS	Genus
52	Cetraria/Dactylina	Lichen	CETDAC	Genus
53	Cladina/Cladonia	Lichen	CLADIDO	Genus
54	Nephroma	Lichen	NEPHRO	Genus
55	Peltigera	Lichen	PELTIG	Genus
56	Stereocaulon	Lichen	STEREO	Genus
57	Unkn Lichen	Lichen	UKNLIC	PFG
58	Aulacomnium Moss	Moss	AULAMO	Genus
59	Classic Moss	Moss	CLASMO	Genus
60	Polytrichum Moss	Moss	POLYMO	Genus
61	Sphagnum moss	Moss	SPHAGMO	Genus
62	Unkn Moss	Moss	UKNMO	PFG
